# SPACE: an algorithm to predict and quantify alternatively spliced isoforms using microarrays

**DOI:** 10.1186/gb-2008-9-2-r46

**Published:** 2008-02-29

**Authors:** Miguel A Anton, Dorleta Gorostiaga, Elizabeth Guruceaga, Victor Segura, Pedro Carmona-Saez, Alberto Pascual-Montano, Ruben Pio, Luis M Montuenga, Angel Rubio

**Affiliations:** 1 CEIT and TECNUN, University of Navarra, San Sebastián, Spain; 2 Integromics SL, Madrid, Spain; 3 Computer Architecture Department, Facultad de Ciencias Físicas, Universidad Complutense de Madrid, Madrid 28040, Spain; 4 Center for Applied Medical Research, University of Navarra, Pamplona, Spain; 5 Department of Biochemistry, University of Navarra, Pamplona, Spain; 6 Department of Histology and Pathology, University of Navarra, Pamplona, Spain

## Abstract

Exon and exon+junction microarrays are promising tools for studying alternative splicing. Current analytical tools applied to these arrays lack two relevant features: the ability to predict unknown spliced forms and the ability to quantify the concentration of known and unknown isoforms. SPACE is an algorithm that has been developed to (1) estimate the number of different transcripts expressed under several conditions, (2) predict the precursor mRNA splicing structure and (3) quantify the transcript concentrations including unknown forms. The results presented here show its robustness and accuracy for real and simulated data.

## Abstract 2

SPACE is an algorithm developed to predict and quantify the pre-mRNA splicing structure of transcripts using exon and ‘exon plus junction’ microarray data.

## Background

Alternative splicing (AS) is the process by which multiple mature mRNA sequences can be generated from the same precursor mRNA (pre-mRNA) upon the differential joining of exonic sequences limited by 5' and 3' splice sites. Through splicing mechanisms exons can be extended or shortened, skipped or included, and intronic sequences may even be retained in the mRNA sequences. AS is one of the most important sources of protein diversity in vertebrates, and at least half of human genes are alternatively spliced [1-3]. AS has been shown to be very relevant in a variety of human diseases, including cancer, and there is increasing interest in the use of AS in developing diagnostic tools and identifying new therapeutic targets [4-7].

Two main strategies are pursued to identify and characterize AS events in expressed genes under both physiological and pathological conditions. On the one hand, expressed sequence tag (EST) alignment and mapping against known proteins or the whole genome may be used to identify different mRNA isoforms expressed in cell lines or tissues [8]. On the other hand, by performing analyses of splicing microarrays, the detection of new isoforms of a gene [9] and quantification of the relative concentrations for known isoforms may be obtained [10].

The most important manufacturers of commercial array platforms intended for the analysis of the expression of alternatively spliced isoforms are Affymetrix, Jivan Biotechnology (based on Agilent technology) and Exonhit (which can work both with Affymetrix and Agilent technologies). The strategy for Jivan and Exonhit includes two types of probes: exon probes or junction probes. Affymetrix uses only exon probes. Exon probes are complementary sequences to each of the known transcribed exons of a given gene, while junction probes include a segment of complementary nucleotides for each of the two sides of a known exon-exon junction in the mature mRNA of the gene. When designing expression arrays, exon probes are usually selected to meet a number of quality criteria. Since these probes may be located anywhere within the exon, probe specificity and affinity can usually be optimized to maximize their performance in the array hybridization step. In contrast, the selection for junction probes has very little room for optimization, as the nucleotide sequence at both sides of the junction is fixed and needs to be included in the junction probe. The only way to optimize junction probe quality is by changing the number of nucleotides at either side of the junction, and thus the total length of the probe. The overall performance of junction probes in any array is therefore remarkably lower than exon probes owing to poorer signal, frequent cross-hybridization, etc. This suboptimal probe quality leads to large variations in the signal levels for different probes of the same transcript, which may differ by as much as several orders of magnitude. Furthermore, the obvious phenomenon of cross-hybridization of a single probe with different transcripts that share half of the probe contributes to make the interpretation of junction-based arrays a real challenge [11]. A potential way to overcome this hurdle is to examine the probes mapped to a gene as a whole instead of analyzing individual probes one at a time.

Several tools and strategies have been proposed to deal with the complex bioinformatics analysis of splicing microarrays [9-16]. Cuperlovic-Culf *et al.*[17] provide a good and up-to-date comparative review of the traits and the performance of each available tool. Three of these strategies [9,13,15] can predict the existence of novel isoforms, but none of them is able to infer the intron/exon structure of the gene. In addition, only three of the previous works [10,12,14] provide a method to measure the relative concentrations of known transcripts. The aim of the present study is to develop a tool to measure the concentrations and structure of different transcripts from the output data of splicing microarrays (containing exon probes together with junction probes). The algorithm we propose here, which we have called SPACE (splicing prediction and concentration estimation), can (1) predict the number of different transcripts (some of them possibly unknown), (2) predict the structure of these transcripts and (3) measure their relative concentrations.

Our algorithm applies 'non-negative matrix factorization' (NMF) to the matrix of data. NMF is a factorization for non-negative multivariate data that allows us to find parts-based linear representations [18]. The main characteristic of NMF is its use of non-negative constraints. Given a matrix of non-negative data *V*, NMF finds an approximate factorization *V *≈ *W*·*H *into matrices with non-negative elements *W *and *H *[19]. In this work, we show that, when applied to splicing microarray data, NMF separates the data matrix for each gene into the product of two positive components corresponding to the structure of the gene transcripts and their individual concentrations, respectively. We have also developed an algorithm to determine the internal dimension of the factorization since previous attempts by other groups did not perform well in this particular application. We show that the internal dimension of the factorization is an estimate of the number of transcripts of each gene.

In summary, SPACE allows for the discovery of the structure of the expressed transcripts of a given gene, as well as for the determination of the relative concentration of each spliced isoform. It also makes the prediction/detection of new, previously unknown, alternatively spliced forms possible.

## Results and discussion

We have applied the NMF algorithm described in the materials and methods section to both synthetic and real microarray datasets. For each gene, NMF of the expression matrix is performed *V *≈ *W*·*H*.

Here *H *gives the relative concentration of each transcript while *W *gives the gene structure, that is, which probes hybridize to each transcript. The mathematical model used shows that the maximum value of each row of the *W *matrix is the affinity of the corresponding probe. Using this information it is possible to discern whether a probe hybridizes against a transcript (the corresponding entry of the *W *matrix is close to the row maximum) or not (the entry is close to zero).

### Synthetic dataset

We have prepared a synthetic dataset to test the NMF algorithms. We generated this dataset as follows. Probe expression is proportional to the sum of concentrations of the transcripts that share the probe. The proportional constant, that is, the affinity, is a random number obtained from a distribution that mimics the distribution of real microarray data. Transcript concentrations are also random numbers. For junction probes, we simulated that these probes partially hybridize (20%) with each transcript that shares one of the sides of the junction. Hybridization is complete (100%) if the (exon or junction) probe matches perfectly with the transcript.

Among the possible parameters to consider while testing the algorithm, we have selected three: (i) noise level (five levels of additive noise and five levels of multiplicative noise); (ii) number of microarrays (5, 10, 25, 50, 100 and 200 arrays); (iii) position of the probes (only junctions J, only exons E, both of them J_E, junctions plus several probes per exon J_2E, J_3E). To make the simulation closer to a biological reality, we have borrowed the structure of eight different genes (CASP2, HNRPA2B1, BCL2L1, BIRC5, TERT, VEGF, BAX and WT1). These genes were selected just as examples and only by structural criteria among a larger list of genes whose AS is changed in cancer. Using the structure of these genes, simulated affinities, simulated concentrations, random noise and assuming a small amount of cross-hybridization for junction probes we have built the simulated expression data matrix. Table 1 shows the basic structural characteristics of these genes. The specific splicing structure is shown in Additional file 1 (Figures S1-S8).

**Table 1:** Description of the synthetic dataset genes used to test the SPACE algorithm

**Gene name**	**Transcripts**	**Exons**
CASP2	2	12
HNRPA2B1	2	12
BCL2L1	2	3
BIRC5	3	4
TERT	4	16
VEGF	4	8
BAX	4	6
WT1	4	10

The basic structural characteristics (number of transcripts and number of different exons) of the genes used to generate the synthetic dataset are shown. The SPACE algorithm has been tested with these genes.

Each simulation has been run 200 times (we tested 20 different combinations of probe affinity and transcript concentrations and each combination was run 10 times in the background of different random noises). The NMF iteration was run 3,000 times for each point to achieve convergence.

**Figure 1:**
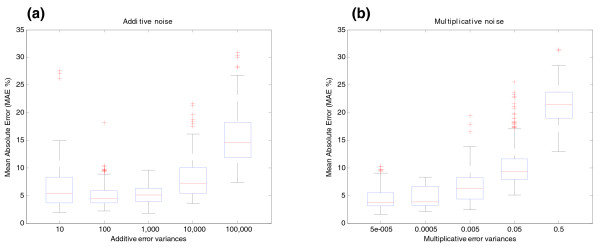
Influence of noise on the estimation of relative transcript concentrations of BIRC5 gene (synthetic data). BIRC5 gene structure is shown in Figure 6a and in Additional file 1 (Figure S4). **(a)** Additive noise effect on estimation of relative transcript concentrations. The *y*-axis shows the MAE between the relative concentration of transcripts without noise and that estimated by the algorithm under the effect of different degrees of additive noise (MAE %). Additive noise is in the form of *y *+ *ε *with *ε *~*N *(0, *σ*_*ε*_^2^). The units of the *x*-axis are the variances *σ*_*ε*_^2 ^of the additive error added to the simulated concentrations (10, 100, 1,000, 10,000, 100,000). These variances represent roughly 0.5%, 2%, 5%, 15% and 50% of the energy of the signal, respectively. **(b)** Multiplicative noise effect on estimation of relative transcript concentrations. The *y*-axis shows the MAE between simulated and estimated relative concentrations under the effect of different degrees of multiplicative noise (MAE %). Multiplicative noise is in the form of *y*·*e*^*η *^with *η* ~*N *(0, *σ*_*η*_^2^). The units of the *x*-axis represent the variances *σ*_*η*_^2 ^of the multiplicative error (5 × 10^-5^, 0.0005, 0.005, 0.05, 0.5). These variances represent roughly 0.7%, 2%, 7%, 25% and 100% of the energy of the signal, respectively. The different degrees of additive and multiplicative noise are tested while the other parameters are in the 'central point' condition (40 arrays and probes at exons and junctions). This means that there is always a component of additive and multiplicative noise in the form of *y*·*e*^*η *^+ *ε*. Errors are represented by boxplots. A boxplot is a graphical representation of the variability of a random signal. They are composed by a box and a whisker. The box extends from the lower quartile to the upper quartile values and there is an additional horizontal line that shows the median. The whiskers are vertical lines extending from each end of the boxes to show the extent of the rest of the data. Outliers are data with values beyond the ends of the whiskers and are represented by crosses.

**Figure 2:**
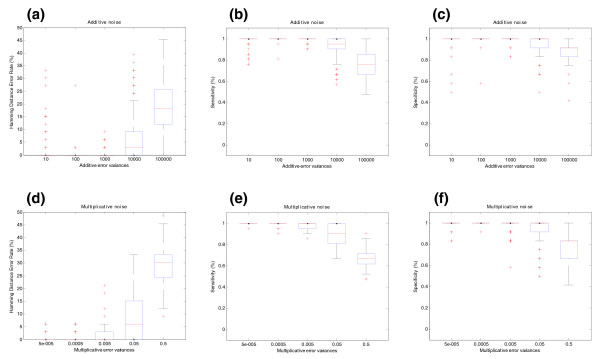
Influence of noise on splicing structure prediction for BIRC5 gene (synthetic data). **(a)** The effect of additive noise on splicing structure prediction. The *y*-axis shows the Hamming distance error rate between real and predicted pre-mRNA splicing structures. This measure represents the proportion of probes in a gene that were mistakenly assigned (or unassigned) to each transcript. The units of the *x*-axis are the variances *σ*_*ε*_^2 ^of the additive error as explained in Figure 1a. **(b) **Sensitivity of the SPACE algorithm under additive noise. Sensitivity is defined as the proportion of probes that belong to each transcript that are correctly assigned in the predicted structure. **(c)** Specificity of the SPACE algorithm under additive noise. Specificity is defined as the proportion of probes that do not belong to a particular transcript that are correctly unassigned in the predicted structure. **(d)** Multiplicative noise effect on splicing structure prediction. The *y*-axis shows the Hamming distance error rate between real and predicted pre-mRNA splicing structures. The units of the *x*-axis are the variances *σ*_*η*_^2 ^of the multiplicative error as explained in Figure 1b. **(e)** Sensitivity of SPACE under multiplicative noise. **(f)** Specificity of SPACE under multiplicative noise. The Hamming distance error rate is calculated in the form of *HD *= (*FP *+ *FN*)/*N*, the sensitivity is calculated as *SN *= *TP*/(*TP *+ *FN*) and the specificity is calculated as *SP *= *TN*/(*TN *+ *FP*).

This study confronted us with a formidable problem: if each individual combination of parameters was checked, the number of conditions would be enormous (five additive noise levels × five multiplicative noise levels × six different numbers of arrays × five probe selections × eight genes = 6,000). These conditions should then each be simulated 200 times, and for each one we would need 3,000 iterations. In order to avoid this problem, we selected a 'central point' for each of the parameters and only one parameter was changed for each simulation experiment to analyze its effects. The 'central point' was selected with the following conditions: standard deviation of additive error was 5% of the median of the signal, standard deviation of multiplicative error was 7% of the signal, number of arrays was 40 and probes were located at exons and junctions (J_E). We performed these simulation experiments on the gene BIRC5 (results for other genes are similar, data not shown). We selected the mean absolute error (MAE) to measure the quality of the concentration estimation and the average Hamming distance to measure the quality of the predicted structure. We defined the Hamming distance as the proportion of probes in a gene that were mistakenly assigned (or unassigned) to a transcript. In Figures 1 and 2 we show an example of these simulations for gene BIRC5, a gene with five exons and three alternative transcripts. We now discuss the results of this prediction. We show the results only for the gene BIRC5, but the overall trend in the other genes is similar.

Additive and multiplicative error are well rejected by the algorithm (Figure 1). Only for very large variances does the quantification have large errors (MAE of the expression matrix for large variances is about 20%). The structure of the gene is correctly predicted in almost any case for low variances as shown in Figure 2. This figure also shows that the median of the Hamming distance is null for small variances, that is, each probe is perfectly assigned to each transcript for most of the simulations. The boxplots in panels B and C display the specificity and sensitivity of the SPACE algorithm for additive error. Both specificity and sensitivity have been calculated using a threshold of 0.5, that is, a probe is considered to belong to a transcript if its entry in the *G *matrix is larger than 0.5 and is not considered to belong to the transcript if the entry is smaller than 0.5. These figures show that both specificity and sensitivity are very good for small errors, and their performance worsens for larger errors. The same applies to multiplicative error in panels E and F.

**Figure 3:**
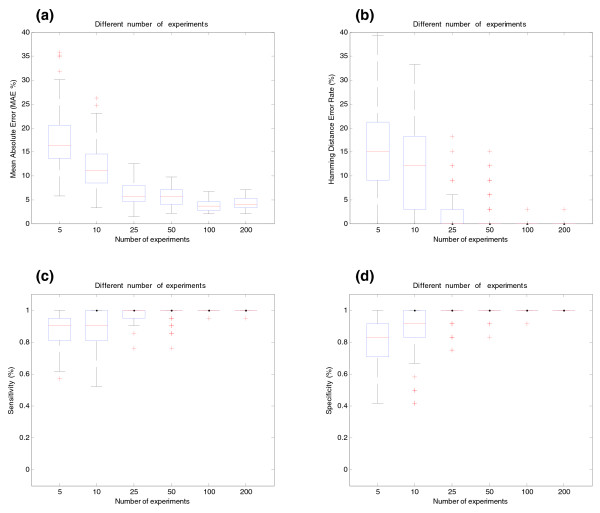
Influence of the number of arrays for BIRC5 gene (synthetic data). **(a)** Effect of the number of arrays on estimation of relative transcript concentrations. The *y*-axis shows the MAE (%) between simulated and estimated relative concentration of transcripts. The *x*-axis shows the different number of arrays used in the simulations. **(b)** Effect of the number of arrays on splicing structure prediction. The *y*-axis shows the Hamming Distance Error Rate between real and predicted pre-mRNA splicing structures. **(c)** Sensitivity of SPACE under different numbers of arrays. **(d)** Specificity of SPACE under different number of arrays.

Figure 3 shows the different results (for gene BIRC5) obtained when the number of arrays per experiment is changed. As expected, the structure is better estimated as the number of arrays per experiment increases. As a side effect, concentration estimation is also improved (Figure 3a).

**Figure 4:**
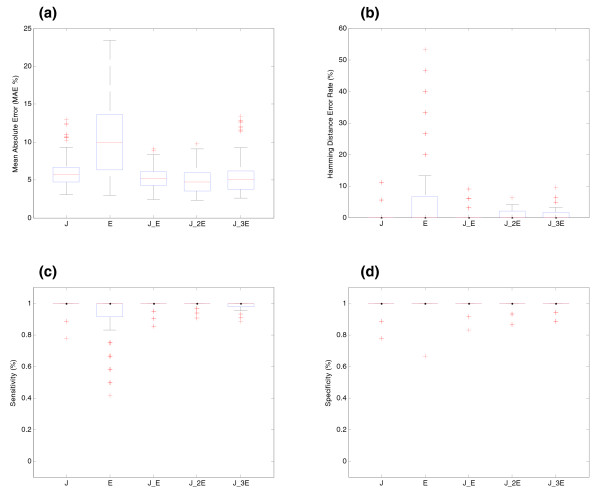
Effect of the location of probes for BIRC5 gene (synthetic data). **(a)** Effect of the location of the probes on the estimation of relative transcript concentrations. The *y*-axis shows the MAE (%) between simulated and estimated relative concentration of transcripts. The *x*-axis shows the different location of probes along the transcripts of the gene. J: the gene is represented by all its junction probes; E: the gene is represented by exon probes located in all its exons; J_E: all junction and exon probes are present in the array; J_2E or J_3E: all junctions and two or three probes per exon, respectively, are present in the array. **(b)** Effect of the location of the probes on splicing structure prediction. The *y*-axis shows the Hamming distance error rate between real and predicted pre-mRNA splicing structures. **(c)** Sensitivity of SPACE with varying location of the probes. **(d)** Specificity of SPACE with varying location of the probes.

Figure 4 shows the results for experiments changing the number and type of probes for BIRC5. Adding new exon probes does not significantly improve the performance of the concentration estimation but helps to estimate the structure of the transcripts. Junctions probes seem to be more informative than exon probes in this simulation. Nevertheless, this result should be taken cautiously since junction probes tend to be of inferior quality. This probe quality factor has not been taken into account in generating these synthetic data.

**Figure 5:**
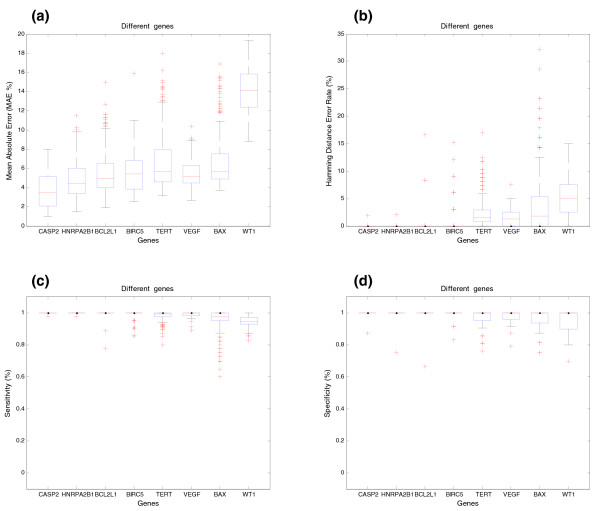
Influence of the gene structure and number of expressed transcripts in a comparative splicing analysis between different genes (synthetic data). **(a)** Estimation of the relative transcript concentrations for different genes. The *y*-axis shows the MAE (%) between simulated and estimated relative concentration of transcripts. The *x*-axis shows the different genes used in the simulation (CASP2, HNRPA2B1, BCL2L1, BIRC5, TERT, VEGF, BAX and WT1). The structure of the different transcripts of these genes and the location of probes is shown in Additional file 1 (Figures S1-S8). **(b)** Prediction of the splicing structure for different genes. The *y*-axis shows the Hamming distance error rate between real and predicted pre-mRNA splicing structures. **(c)** Sensitivity of SPACE for different genes. **(d)** Specificity of SPACE for different genes.

The simulation results when comparing different genes are particularly interesting (Figure 5). These simulations have been performed using the 'central point'. It can be seen that the accuracy of structure prediction decreases with the number of transcripts (see Table 1). For the same number of transcripts, structure predictive accuracy increases by increasing the number of probes with different hybridization patterns. The hybridization pattern is defined as the binding capability of a probe with each of the transcripts of a gene, that is, a logical vector that shows whether the probe belongs to each transcript or not.

**Figure 6:**
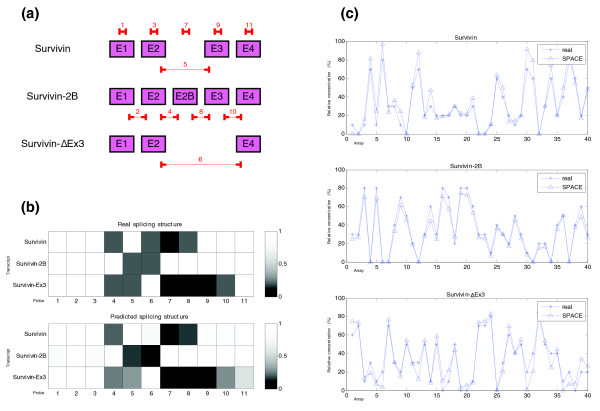
Predicted structure and estimated concentrations for the BIRC5 (apoptosis inhibitor survivin) gene in the 'central point' (synthetic data). **(a)** Structure of the different transcripts of the BIRC5 gene and location of probes used in the simulation. **(b)** Representation of the real and predicted splicing structures for the BIRC5 gene given by the probes used. In the graphic representing the real splicing structure the probes that match perfectly with the transcripts are represented by a white box (100% matching) and no hybridization is shown by a black box (0% matching). Gray levels show intermediate matching values. We have assumed that junction probes which include one side of the junction hybridize partially (20%). **(c)** Estimated relative concentrations of the three isoforms of BIRC5 gene. In each of the three graphics simulated and estimated relative concentration of each isoform is represented.

The predicted and real structures and concentrations for genes that have an error close to the median of the simulations are shown in Figure 6. The comparison between real and predicted structure of BIRC5 (Figure 6b) clearly shows an almost perfect matching.

**Figure 7:**
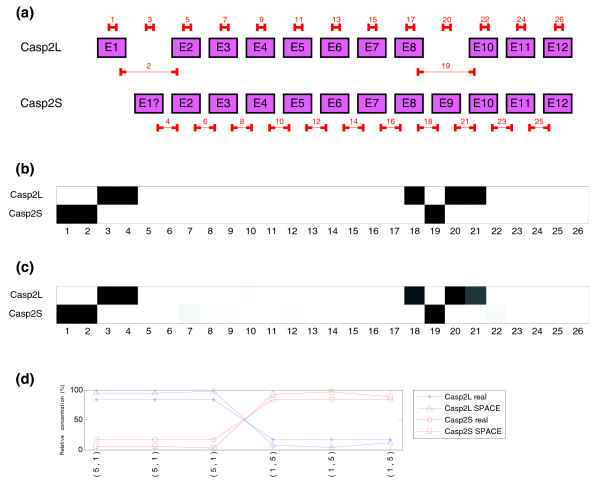
Experiment done with the CASP2 gene, transcripts Casp2L and Casp2S (synthetic data). Three arrays were performed with a concentration ratio between the two isoforms of CASP2 gene equal to 5:1 and another three with the opposite ratio 1:5. **(a)** Structure of the two transcripts of CASP2 gene and location of probes in the microarray. **(b)** Real structure of CASP2 gene indicated by probes. Probes that match perfectly are represented in white (100%), no hybridization in black (0%) and partial hybridization by different shades of gray (20%). **(c)** Predicted splicing structure for CASP2 gene with the alternating concentration ratio 5:1. If compared with the real structure of CASP2 transcripts (b), a strong similarity is noticed. **(d)** Real and estimated relative concentrations of the two isoforms of CASP2 gene in the experiment.

To further study the limits of the algorithm we provide a simple simulation: an experiment with gene CASP2 (that has two transcripts Casp2L and Casp2S) in which three arrays have been constructed under one condition and three arrays under a second condition. The number of arrays is low and quantification errors, as Figure 7d shows, are larger than in the central point. Since this study is focused on AS, we considered that the overall concentration of the gene is constant (that is, the sum of the concentrations of the transcripts), and we have changed the relative abundance of each isoform. This sort of change cannot be detected by an expression array. We performed three different experiments in which the concentration ratio between each condition was 5:1 (Figure 7), 2:1 and 1.5:1 (see Additional file 1, Figure S10). These experiments show that splicing structure degrades as concentrations become closer to 1:1.

To improve the robustness of the performance figures, we have randomly selected 100 genes from the human genome with transcripts that range from 2 to 5. The number of exons of the selected genes ranges from 1 to 74.

We generated synthetic data for these genes assuming similar conditions to those selected for the central point: (1) junction probes and exon probes are included, (2) certain partial hybridization occur with junction probes (20%) and (3) additive and multiplicative noise with the same variance of the noise used in BIRC5 have been simulated.

A summary of the results obtained for these genes is shown in Table 2. In this table, the median value of each error measurement is shown in bold face between the lower and upper quartiles for equal number of transcripts. It can be noticed that the median values of MAE, Hamming distance, sensitivity and specificity corroborate the general trend found in the selected genes that performance decreases if the number of transcripts per gene increases. A more detailed description of the simulation results is available in Additional file 1 (Figures S11-S22).

**Table 2:** Summary of simulation results for 100 random genes (synthetic data)

**Number of transcripts**	**MAE**	**Hamming distance**	**Sensitivity**	**Specificity**
**2**	3.1%-**4.6%**-7.0%	0%-**0%**-0%	1 - **1 **- 1	1 - **1 **- 1
**3**	3.7%-**5.1%**-6.8%	0%-**0%**-0.85%	0.99 - **1 **- 1	1 - **1 **- 1
**4**	4.1%-**5.2%**-7.0%	0%-**1.4%**-2.9%	0.96 - **0.99 **- 1	1 - **1 **- 1
**5**	4.0%-**5.4%**-8.3%	1.5%-**3.4%**-9.6%	0.88 - **0.95 **- 0.98	0.96 - **0.99 **- 1

The median value of MAE, Hamming distance, sensitivity and specificity for the simulation performed with 100 random genes is shown in bold for an equal number of transcripts. The variability of median values of each error measurement are indicated by the lower and upper quartiles at both sides of the median. It should be noted that the error increases as more transcripts are added to the simulation.

### Real datasets

To analyze the performance of the algorithm against biological data, we have tested SPACE on real datasets from large series of microarray hybridization experiments. We used two sets: one generated from an experiment using the Affymetrix platform (Wang dataset) [10] and another generated from an Agilent platform (Johnson dataset) [9].

The Wang dataset used Affymetrix technology to quantify the relative concentration of two transcripts of the CD44 gene. A large number of probes (184 perfect match (PM) and an equal number of mismatch (MM) probes) were available for this gene. On the other hand, in the Johnson dataset Agilent technology was used to monitor the junctions of 10,000 multi-exon genes across 52 diverse samples. In this dataset, we applied SPACE to the analysis of those genes whose splicing prediction was validated by Johnson *et al.* using reverse transcription polymerase chain reaction (RT-PCR).

#### Wang dataset

**Figure 8:**
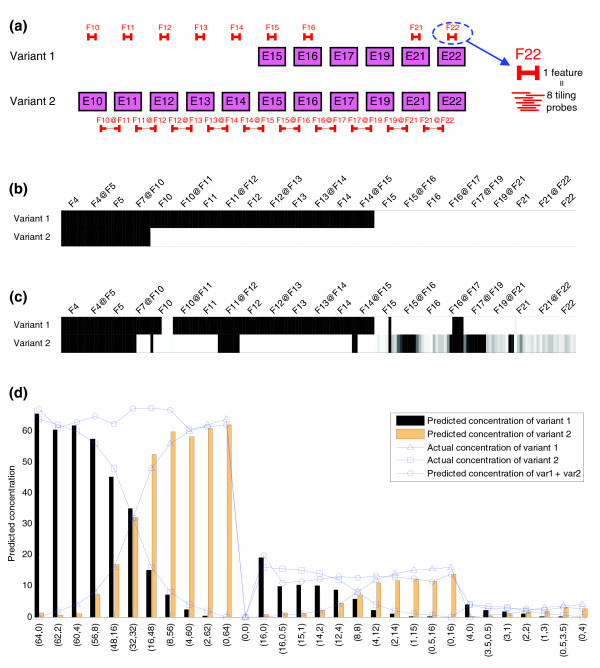
Predicted structure and estimated spiked concentrations for the CD44 gene. **(a)** Structure of the CD44 gene spiked transcripts and probe positions represented by features. A gene feature is either an exon or a junction. Exon features are represented by F followed by the number of the corresponding exon and junction features are represented by the two exon features to which they belong joined by @ symbol. Each feature is made up of eight probes following a tiling strategy. As 23 features have been measured this makes a total of 184 probes. Probes corresponding to exon features F4, F5 and junction features F4@F5, F7@F10 do not match any of the spiked transcripts and therefore are not shown in (a). **(b)** Expected hybridization pattern of all probes for each of the two variants of CD44 gene. **(c)** Splicing structure prediction for CD44 gene applying the SPACE algorithm. **(d)** Estimated concentrations of the two variants of CD44 gene compared to spiked concentrations. The *y*-axis shows the predicted and actual concentration of each variant. The *x*-axis indicates the experiments and actual concentrations of each variant pair.

In this set, we used the 184 PM probes to measure two transcripts of CD44 spiked by Wang *et al.* in their experiment [10]. Even though we performed the simulations using two transcripts, we noticed that results assuming three transcripts were better. Results with three transcripts are shown in Figure 8. After performing the factorization, we noticed that the entries of the row of the *W *matrix corresponding to the third transcript had almost the same constant value. We concluded that our algorithm is mimicking the proposed method in [10] to remove background noise. Concentrations of the two spiked transcripts are well estimated (MAE = 4.47%). The structures of the transcripts are well predicted except for junctions 7-10, 11-12 and 16-17 and for exon 10 (which is incorrectly included in the first transcript by the algorithm).

We have tried the algorithm with other parameters (using two transcripts and the difference between PM and MM measurements, three transcripts using both PM and MM probes) and results are similar.

Surprisingly, when we considered only MM probes for the algorithm, it was also possible to predict the concentration of the transcripts with worse yet still reasonable accuracy (MAE = 14.06%). Even though our results are similar to Wang *et al.*'s (in fact, their results are so good that there is little room for improvement), we obtained them without any *a priori *knowledge of gene structure. Therefore, the relevant and major contribution of SPACE is that it can estimate concentration without knowing the structure of the gene. In addition, if part of the structure is known it can be readily included in our algorithm, as will be stated in the discussion. SPACE can be considered as a generalization of Wang *et al.*'s algorithm that is able to both predict structure and concentration of transcripts and is also able to take advantage of partial knowledge of the structure.

#### Johnson dataset

The analysis performed by Johnson *et al.* to interpret their data can be considered as a variation of an analysis of variance (ANOVA) type II test using medians instead of means. For each log transformed expression, the median of probe expressions in different tissues (to remove the affinity effect) and the median of probe expression in each gene (to remove the gene level effect) were subtracted to obtain a residual. In a second step, they performed a discretization of the residues. Genes with large residues for several tissues are further analyzed since these patterns are probably due to AS.

As explained in the introduction, junction probes cannot meet quite so stringent quality criteria as exon probes. In addition, the number of probes per gene is much smaller than in the Wang dataset.

Furthermore, this dataset was obtained from real tissues instead of spiked transcripts. Therefore the results are not expected to be as accurate as in the previous dataset. The relative concentrations of transcripts were predicted using the SPACE algorithm. In the following we briefly describe the results for each gene and discuss the performance of our algorithm when compared with the RT-PCR analyses.

The first analyzed gene is OCRL (ENSG00000122126). Two splice variants were detected using RT-PCR in Johnson *et al.*'s paper. Therefore, we used an internal dimension of two transcripts to apply the algorithm.

**Figure 9:**
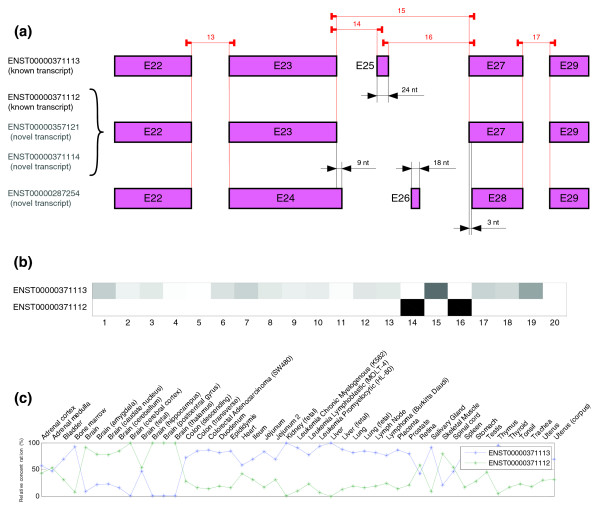
Predicted structure and estimated relative concentrations for the OCRL gene. **(a)** Structure of the different transcripts (known and novel) of OCRL gene according to Ensembl 40, as well as the real location of the probes in the microarray. As can be seen in the figure, given probes cannot distinguish between a group of three isoforms (one known and two novel). **(b)** Predicted splicing structure for OCRL gene given by probes. The SPACE algorithm only detect two isoforms that match with known transcripts of OCRL gene ENST00000371113 and ENST0000037112. **(c)** Estimated relative concentrations of the two isoforms detected of OCRL gene.

Estimated relative concentrations (Figure 9c) match Johnson *et al.*'s expression results obtained by RT-PCR. Comparing the colormap shown in Figure 9b with the gene structure (for Ensembl release 40) shown in Figure 9a, it is possible to infer that the variant 1 predicted by the algorithm corresponds to ENST00000371113 and variant 2 corresponds to a group of three transcripts (the set of probes included in the array is not able to distinguish among the three of them). In these cases, the structure predicted by SPACE matched that of Ensembl known transcripts. The colormap structure suggests the existence of a cassette splicing event for exon 25 in ENST000037112.

**Figure 10:**
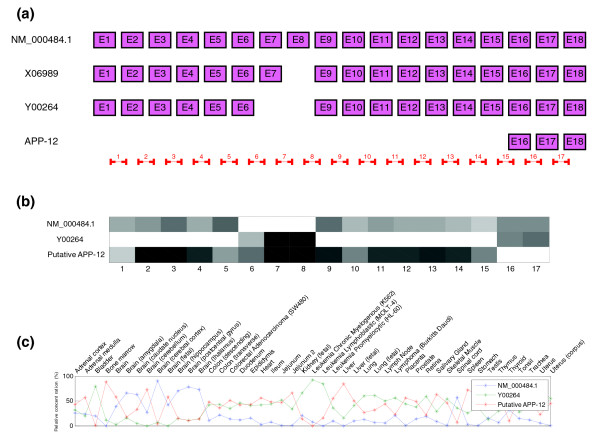
Predicted structure and estimated relative concentrations for the APP gene. **(a)** Structure of the three different transcripts of the APP gene proposed by Johnson *et al*. to be present in the samples as well as a short isoform APP-12 that match our results, the real locations of the probes in the microarray are also indicated. **(b)** Predicted splicing structure for the APP gene given by probes. SPACE detects three isoforms that match with transcripts NM_000484.1, Y00264 and APP-12 of the APP gene. **(c)** Estimated relative concentrations of the three isoforms detected for the APP gene.

Results for APP gene (ENSG00000142192) are shown in Figure 10. Estimated relative concentrations using three transcripts match Johnson *et al.*'s PCR results. The structure prediction is less accurate in this case. According to Johnson *et al.*, this gene has three transcripts: a long form, one with a single exon cassette (exon 7) and one with a double exon cassette (exons 7 and 8). The algorithm is able to predict the structure of the long isoform and the double cassette isoform correctly but not the single cassette isoform. The third form shows what seems to be a splicing event related to probes 16 and 17, the 3' region of the gene. VEGA (a curated genome database) shows that there is a short transcript (APP-012) that has experimental evidence and involves precisely these two probes. This result may be a coincidence but a careful study of these probes shows that they do not correlate well with the others, suggesting either a splicing event or an artifact of the array. The estimated concentrations of the long and double cassette isoforms match the results obtained by PCR in the original article.

**Figure 11:**
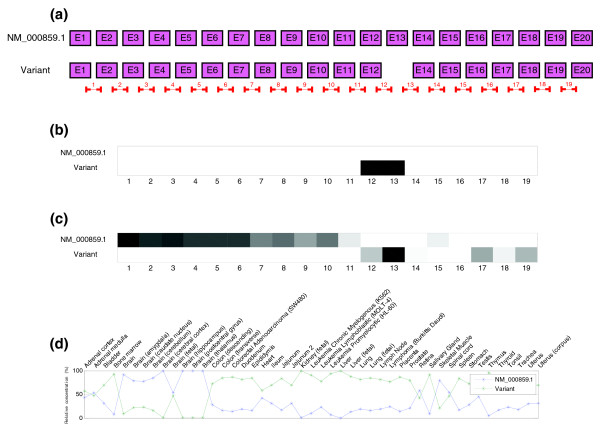
Predicted structure and estimated relative concentrations for the HMGCR gene. **(a)** Structure of the two transcripts of the HMGCR gene, NM_000859.1 and a variant with a cassette in exon 13, as well as the real locations of the probes in the microarray. **(b)** Predicted splicing structure for the HMGCR gene given by probes. If compared with the gene structure in (a), it can be seen that the cassette is detected but also more things that do not match with that model. **(c)** Real and estimated relative concentrations of the two isoforms of HMGCR gene.

Results for HMGCR gene (ENSG00000113161) using two transcripts are shown in Figure 11. In this case, the estimation of the concentrations is not as clear as in the other genes. Indeed, PCR concentrations in Johnson *et al.*'s paper show only significant differences in the concentrations for a tissue that was not hybridized in the set of arrays (peripheral leukocytes). Structure prediction shows the expected results (exons 12 and 13, where there is a cassette, have the smallest affinities along the probes for variant 2).

### Prediction of number of transcripts

In the previous sections we have shown the potential of SPACE in determining the concentration and splicing structure of the genes, assuming that the number of transcripts is known. However, when the number of transcripts is unknown, this method can also be used to make a accurate prediction. In this section we describe this novel application using the synthetic and real datasets.

We have estimated the number of transcripts using the algorithm presented in the materials and methods section. In brief, this algorithm searches for the dimension that optimally splits the error figure for different numbers of transcripts into two groups: signal and noise.

The estimated number of transcripts for the initial list of eight genes used in the synthetic dataset is shown in Table 3. The estimated number is correct for all of the genes except WT1. In this case, the algorithm predicts three transcripts (instead of four) in 100% of the simulations. If WT1 structure is analyzed, it can be seen that two of their transcripts share all but one of the probes. At this level of noise, the algorithm finds it more likely to have three transcripts than four. The estimation of the number of transcripts improves dramatically for the other genes since they have a larger number of probes with different hybridization patterns.

**Table 3:** Prediction of number of transcripts per gene (synthetic data)

	**Number of transcripts**					
**Gene name**	**1**	**2**	**3**	**4**	**5**	**6**

CASP2	0%	**100%**	0%	0%	0%	0%
HNRPA2B1	0%	**95%**	5%	0%	0%	0%
BCL2L1	0%	**90%**	10%	0%	0%	0%
BIRC5	0%	0%	**95%**	5%	0%	0%
TERT	0%	0%	10%	**85%**	5%	0%
VEGF	0%	0%	0%	**90%**	10%	0%
BAX	0%	0%	20%	**80%**	0%	0%
WT1	0%	0%	100%	**0%**	0%	0%

To apply NMF factorization, it is necessary to know, or estimate, the number of transcripts of a particular gene present in the samples under study. The estimations of the number of transcripts using SPACE for the genes used in the synthetic dataset are shown. Each entry of the table shows the proportion of predicted number of transcripts for different simulated data. The column corresponding to the real number of transcripts is shown in bold.

**Figure 12:**
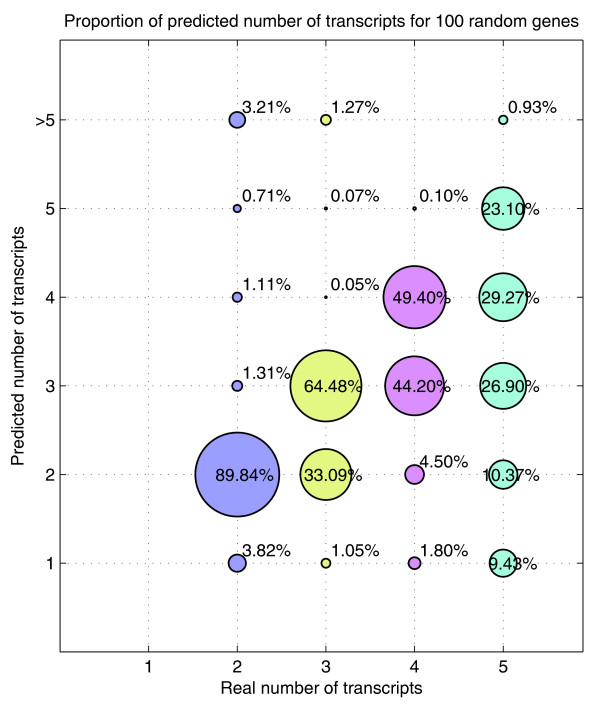
Proportion of predicted number of transcripts for the simulation performed with 100 genes (synthetic data). The 100 genes used have been randomly selected from the human genome with two to five transcripts. Each of the genes have been simulated 200 times for different noise, concentrations and affinities. The area of each circle represents the proportion of times the corresponding predicted number is chosen by the algorithm for a given number of transcripts. The algorithm tends to underestimate the number of transcripts as the real number of transcripts increases.

In Figure 12, we further extend the analysis of predicting the number of transcripts for the simulation performed with 100 random genes of the human genome (synthetic data). This figure shows that the accuracy of the prediction decreases as the number of transcripts increases. It can also be noticed that the algorithm tends to underestimate the real number of transcripts when it does not make a correct guess. We describe two possible reasons that we have found for such a behavior. In some cases, there are very similar transcripts that can be discerned by only one probe (similar to the WT1 case). On the other hand, if some of the transcripts have very low concentrations they are considered to be noise.

The estimation performed in the Wang real dataset predicts three transcripts. As already explained, the SPACE algorithm behaves better using this result as the number of transcripts when compared with using the real number is two (the third transcript is measuring the background noise).

The results for the three genes in the Johnson dataset are as follows. In the OCRL gene (ENSG00000122126), SPACE estimated that two transcripts are present. In the case of the APP gene (ENSG00000142192) two transcripts are predicted (but the likelihood for three transcripts is similar).

Finally, SPACE predicted two transcripts for the HMGCR gene (ENSG00000113161).

## Discussion

We have described a method to predict the number, structure and concentration of gene transcripts using splicing microarray data.

Our simulations show that the SPACE algorithm method is able to predict unknown structures and to measure the relative concentration of alternatively spliced isoforms from synthetic data. The method is robust against multiplicative and additive noise. Structure prediction performance of SPACE increases with the number of arrays.

As expected, performance diminishes with an increasing number of transcripts per gene. The accuracy of the prediction is closely related to the number of probes that have different hybridization patterns along the transcripts. This fact is even more evident when analyzing the results of the simulation: additional exon probes do not improve the discriminative power of the array as much as including probes that provide new hybridization patterns. A good microarray design to detect splicing should have as many discriminative probes per gene as possible. An algorithm was previously proposed to select discriminative probes subject to constraints based on the Hamming distance [20]. This algorithm can be readily applied to splicing arrays.

Our simulation experiments also show that junction probes tend to be better at distinguishing between transcripts than exon probes. In contrast, partial hybridization with adjacent exons does not seem to be a problem. A major drawback associated with these types of splicing array is the poor thermodynamic quality of many junction probes. SPACE is useful in interpreting the output data from splicing arrays containing the usual proportion of suboptimal junction probes. In fact, we have also shown that the SPACE algorithm performs successfully with real splicing microarray datasets. Our analysis of the Wang dataset [10], based on an Affymetrix platform, estimated very similar transcript relative concentrations to those obtained in Wang *et al.*'s paper. Our data were also close to the real concentration of the spikes. Moreover, compared with previous studies, our algorithm provides a method to predict the structure of unknown isoforms. This feature is unique and novel since previous algorithms have only dealt with the probes that may be related to splicing events but do not predict any structure. In addition, our study provides a method to estimate relative concentrations of known and novel isoforms.

The SPACE algorithm deals with all of the probes as a whole and, as shown by the simulations, it is able to reject additive and multiplicative noise. The gene structure predicted by SPACE when using the Johnson dataset [9], based on an Agilent platform, performed better for the genes with two transcripts than for the genes with three transcripts, in which the structure of the third transcript was less accurately estimated. Concentration estimations were similar to those reported in Johnson *et al.*'s paper by means of RT-PCR.

The SPACE algorithm has certain limitations that must be taken into account when applying it. First, it assumes that the probe signal levels have been derived from a linear model. If the probe signals are not proportional to the concentration of the transcripts, both the structure and the concentrations can be predicted incorrectly. Second, we have tested that the factorization works better (that is, error diminishes) if there is variability in *W *and *H*. For the *W *matrix this means that the error figures improve if the array includes several probes that are able to distinguish between different transcripts. For the *H *matrix, the prediction power improves if we include several different experimental conditions. If only one experimental condition is performed (for example, in a design which includes several replicas of a single sample), this algorithm is not able to discover a mixture of transcripts.

The level of noise affects the ability of the algorithm to discover new transcripts. If the concentration of a particular transcript is very low, it may be masked by the noise background, and SPACE, in this case, would not be able to discern this transcript.

We have used the maximum value of a row in the *W *matrix to estimate the affinity of the probe and convert the *W *matrix into the product of matrices *AG*. The maximum value is a statistical operation that is strongly affected by outliers. However, NMF, using Kullback-Leibler (KL) distance, is robust against outliers and it is not likely to have an outlier in the final factor matrices. A side effect of this selection is that a probe does not hybridize with any of the transcripts of the gene, a row of zeroes in the *G *matrix, so this method will give a one in some of them. A way to overcome this problem is to identify whether any of the 'discovered' transcripts is, in fact, background noise and reject the probes in which the computed entries for *G *are closer to the value of the transcript that models the noise. This is the approach the we used for the Wang dataset.

As described in the materials and methods section, since only 15% of the exons in genes with AS are specific for a unique transcript, the algorithm fosters to fill the *G *matrix. Small transcripts that only have a few probes that hybridize on them tend to be predicted incorrectly. Many of the errors in the simulation of 100 genes correspond to genes with small transcripts.

This method can be further improved if some constraints are imposed on the NMF model. For example, if the structure is partially known, its corresponding *W *matrix can be used as an initial value for the optimization. The structure of *W *is maintained along the optimization by the algorithm since the described multiplicative update rule retains the null entries of both the *W *and *H *matrices.

On the other hand, the use of NMF multilayer [21] algorithms is a novel optimization technique that uses several matrices, instead of two, to perform the optimization. All of the matrices in these algorithms have positive entries. The original work of Wang *et al.* proposes the factorization affinities, features, property matrix and concentrations, that is, *Y *= *AFGT *+ *E*. This factorization fits perfectly with a multilayer algorithm. In addition, some of these matrices are sparse (the affinity and the features matrix). Imposing sparsity constraints [22] and null entries may help the algorithm to improve the results.

## Conclusion

In this paper we have presented SPACE, an algorithm based on NMF that is able to predict the number of transcripts, gene structure and concentration of known and unknown versions of splice forms. The validity of the results have been tested in both synthetic and biological data, and SPACE has shown its ability to reject additive and multiplicative noise.

It can be stressed that the algorithm not only predicts the concentration, but also the structure of the genes. This characteristic makes it completely novel.

## Materials and methods

### Probe and gene structure model

In our algorithm, we assume that there is a linear relationship between the intensity of a probe *y *(an exon probe or a junction probe) and the concentration of the targets *x *measured by this probe (as proposed by Li and Wong [23]):

*y *= *a*·*x *+ *e*

where *a *is the affinity of the probe and *e *is an error term.

Let the matrix *Y*_*i *× *j *_be the set of measurements of all the probes included in a gene. Its dimensions are *i *rows (probes included in the gene) and *j *columns (different arrays).

Taking into account that each probe may belong to a particular transcript and not to others, we can extend this model by using a 'property' matrix *G*. Combining the information for different transcripts, we can derive the following:

*Y *= *A*·*G*·*T *+ *E*

where, *A *= (*a*_*i*, *i*_) is an *i *× *i *diagonal matrix of unknown affinities. The matrix *T *= (*t*_*k*, *j*_) represents the concentration of each *k *gene transcript (rows) in the *j *array (columns). The property matrix *G *= (*g*_*i*, *k*_) relates the probes with the different transcripts depending on whether the probe belongs to the transcript or not. This model was proposed by Wang *et al. *[10]. In their paper, an additional matrix of features *F *is included, but it can be avoided without loss of generality. The proposed value for each entry in this case is

$$g_{i,k}=\{\begin{array}{ll} 1 & \text{probe }i\text{ is included in transcript }k\\ 0 & \text{probe }i\text{ not included in transcript }k\\ \alpha & \text{probe }i\text{ partially hybridizes with transcript }k\;(\text{useful for junction probes}) \end{array}$$

In Wang *et al.*'s method, this matrix is binary (that is, if no perfect sequence identity is obtained, it is considered that there is no hybridization). We propose that, since partial hybridization does occur in junction probes, a proper selection of parameter *a *can take this fact into account. The value of *a *(partially hybridized probe) ranges from 0 to 0.6 depending on the length of the probes, their composition and manufacturing of the array [11]. Finally, *E *= (*e*_*i*, *j*_) matrix represents the error term.

### Model fitting and minimization

In expression (2), both *A *(the affinity matrix) and *T *(the concentration matrix) are unknown. A natural way to find these unknowns is to minimize some function of the error term. Wang *et al.* proposed to minimize the sum of the squared differences between the measurements (*Y *matrix) and the estimation (*A*·*G*·*T *matrix) subject to the condition that the unknowns (affinities and concentrations) must be positive as follows:

$$\begin{array}{l} \min(||Y-A\cdot G\cdot T|{|}_{2})\\ \text{subject to }a_{i,i}\geq 0\text{ and }t_{k,j}\geq 0 \end{array}$$

where || ||_2 _is the Fröbenius norm of the matrix (that is, the sum of its entries squared).

This optimization function may be ineffective if it is applied to splicing arrays. Since junction probes cannot be selected to have similar affinities (the position of the probe cannot be arbitrarily selected), their affinities vary by several orders of magnitude. This minimization function is proportional to the error squared and results are skewed to model probes with large affinities. Instead of this error function, we propose to use the Kompass family of divergence functions [24]:

$$D_{\text{Ko}}(Y,AGT)=\sum_{i=1}^{p}\sum_{j=1}^{n}\left(Y_{ij}\frac{Y_{ij}^{\beta -1}-{\left(AGT\right)}_{ij}^{\beta -1}}{\beta (\beta -1)}+{\left(AGT\right)}_{ij}^{\beta -1}\frac{{\left(AGT\right)}_{ij}-Y_{ij}}{\beta }\right)$$

This family has an additional parameter *β *that must be set. It can be easily shown that the dimension of the summation term is $\varepsilon_{ij}^{\beta }$. If *β *= 2, this function reverts into expression (2) and it is the selection of choice for additive Gaussian noise. If *β *tends to zero, it reverts into the Itakuro-Saito entropy (especially useful for multiplicative noise) and if *β *tends to one, it reverts to the KL entropy that can be used for noise that includes additive and multiplicative terms.

### Blind gene structure prediction

The standard non-negative matrix factorization can be directly applied to the matrix *Y*, yielding two matrices *W *and *H*:

*Y*_*ij *_≈ *W*_*ik*_·*H*_*kj *_≈ (*A*_*ii*_·*G*_*ik*_)·*T*_*kj*_

It is straightforward to identify *W *with *AG *and *H *with *T *(the relative concentrations of the *k *transcripts). This assignation is done because of their respective dimensions. There is an intuitive interpretation of NMF for the analysis of splicing: the first matrix represents the predicted structure of the gene (whether a particular probe belongs to a particular transcript or not) and the second is the relative abundance of the transcript.

Each of the matrices in the Wang *et al.* equation (2) has an interesting property: all of the entries are non-negative. In addition, most of the exons (in genes that are alternatively transcribed) are shared by several transcripts, that is, the *G *matrix usually has many ones and few zeros. If the NMF of the *Y *matrix is performed (*Y *= *WH*), non-negativity is ensured (first condition), and using certain algorithms, the *W *(the *AG *factor) matrix has few zero entries (second condition). Therefore, NMF provides a reasonable estimation of *A*, *G *and *T *and, as shown in the simulation, they are indeed very close to the real values of these matrices.

One simple way to obtain the *A *and *G *matrices from *W *is to consider that the affinity of a probe is the maximum value for the corresponding row in *W*. In this case, we obtain

$$\begin{array}{c} a_{ii}=\underset{k}{\max}(W_{ik})\\ G=A^{-1}W \end{array}$$

Here *G *will be a matrix whose entries lie between zero and one. The algorithm to perform the optimization (expressed in Matlab compact form) for the Kompass generalized divergence function [24] is

$$\begin{array}{l} H\leftarrow H.*(W^{\prime }*(V./{\left(W*H\right)}^{2-\beta }))./(W^{\prime }*{\left(W*H\right)}^{\beta -1})\\ W\leftarrow {\left(W.*((V./{\left(W*H\right)}^{2-\beta })*H^{\prime })./({\left(W*H\right)}^{\beta -1}*H^{\prime })\right)}^{1+\alpha_{W}}\\ W\leftarrow W.*\text{diag}\{1./\text{sum}(W,1)\} \end{array}$$

where .* represents element wise multiplication and *α*_*W *_is a small constant (around 0.005) that modifies (increases for positive values and decreases for negative values) the sparsity of matrix *W*. We have used for *β *the value of one (that is, we used KL divergence to perform the estimations).

### Implementation issues

We used Matlab 7.1 with the statistics toolbox on a Pentium IV 3.2 GHz PC. The Matlab code is available as Additional file 2, so that other researchers can validate our calculations. The time required to compute each gene depends on the number of probes, the number of transcripts and the number of arrays. For BIRC5 (11 probes, 3 transcripts and 40 arrays), it took 1.2 seconds to perform the factorization using 5,000 iterations. During the last 4,000 iterations the error function did not change, but we decided to use a large number of iterations to ensure convergence. Proper termination conditions may decrease the computing time by a factor of five (about 0.2 seconds per gene). Using these improvements, the computing time for 25,000 genes is less than 2 hours. The algorithm can be easily computed in parallel in a cluster if a shorter computing time is desired. On the other hand, the estimation of the number of transcripts is a computer-intensive task: the factorization has to be performed twice, for original data and randomized data, for up to 10 transcripts. On average, it takes about 20 times more time to estimate the number of transcripts than to perform a single factorization.

### Dealing with non-uniqueness of the decomposition

**Figure 13:**
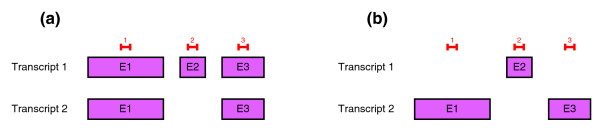
Example of the non-uniqueness of the splicing structure prediction. **(a)** Structure of a generic gene and proposed probe pattern. **(b)** Possible splicing structure prediction obtained by SPACE with the same probe pattern.

Non-negative factorization is not unique: in some cases there are several *W*-*H *pairs that reconstruct the same initial matrix. Other factorizations such as singular value decomposition (SVD) have additional orthogonality constraints that make them unique in most cases (if the singular values are different). When applying NMF to splicing analysis, there can be several structures and concentrations that are compatible with probe measurements for a particular gene. Let us illustrate this fact with an example. Let us assume that (1) all of the probes have the same affinity, (2) the real structure of the transcript is as shown in Figure 13a and (3) two measurements are performed (in the first the concentration of the transcripts are *t*_11 _= 1, *t*_12 _= 0 and in the second *t*_21 _= 0, *t*_22 _= 1).

The matrix equation in this case is

$$\underset{Y}{\underset{︸}{\left[\begin{array}{cc} 1 & 1\\ 1 & 0\\ 1 & 1 \end{array}\right]}}=\underset{A_{1}}{\underset{︸}{\left[\begin{array}{ccc} 1 & 0 & 0\\ 0 & 1 & 0\\ 0 & 0 & 1 \end{array}\right]}}\underset{G_{1}}{\underset{︸}{\left[\begin{array}{cc} 1 & 1\\ 1 & 0\\ 1 & 1 \end{array}\right]}}\underset{T_{1}}{\underset{︸}{\left[\begin{array}{cc} 1 & 0\\ 0 & 1 \end{array}\right]}}=\underset{G_{1}}{\underset{︸}{\left[\begin{array}{cc} 1 & 1\\ 1 & 0\\ 1 & 1 \end{array}\right]}}\underset{T_{1}}{\underset{︸}{\left[\begin{array}{cc} 1 & 0\\ 0 & 1 \end{array}\right]}}$$

This gene structure and concentrations give the matrix of measurements shown in the equation. Let us consider the alternative structure shown in Figure 13b.

In this case, one possible matrix equation for the same measurements could be

$$\underset{Y}{\underset{︸}{\left[\begin{array}{cc} 1 & 1\\ 1 & 0\\ 1 & 1 \end{array}\right]}}=\underset{G_{2}}{\underset{︸}{\left[\begin{array}{cc} 0 & 1\\ 1 & 0\\ 0 & 1 \end{array}\right]}}\underset{T_{2}}{\underset{︸}{\left[\begin{array}{cc} 1 & 0\\ 1 & 1 \end{array}\right]}}$$

or, in a more general case,

$$\underset{Y}{\underset{︸}{\left[\begin{array}{cc} 1 & 1\\ 1 & 0\\ 1 & 1 \end{array}\right]}}=\underset{G_{2}}{\underset{︸}{\left[\begin{array}{cc} 1-\alpha & 1\\ 1 & 0\\ 1-\alpha & 1 \end{array}\right]}}\underset{T_{2}}{\underset{︸}{\left[\begin{array}{cc} 1 & 0\\ \alpha & 1 \end{array}\right]}}$$

It can be seen that the same probe expression matrix *Y *is compatible with two different combinations of properties and concentrations (*G*_1 _and *T*_1 _versus *G*_2 _and *T*_2_). Probe expression does not provide sufficient information to decide between the two versions. However, although it is necessary to determine which of the two versions is real, probe expressions do not provide the necessary information for this.

However, if the data belong to the same gene, it is more likely that an exon is shared by several transcripts. In Ensembl release 40, only 15% of the exons of alternatively spliced genes are specific for a unique transcript. We have computed this number by performing a query against the Ensembl 40 MySQL database. Therefore, if two structures are valid, the structure that shares more exons among transcripts is preferable. From the point of view of *G *matrices, it is preferable to have a full matrix (probes are shared by many transcripts) rather than a sparse matrix (probes are specific to a few transcripts).

NMF factors tend to be sparse (in this example, the NMF factorization will probably give the structure shown in Figure 13b instead of the first). In terms of the factorization, it is necessary to ease the filling of the matrix *W*. This can be done by adding a penalty term related to the sparseness of matrix *W *or, as proposed in [24], selecting the *α*_*W *_parameter in (6) to be a small negative value. We have selected -0.005 as proposed in [24].

### Getting the number of transcripts

There is still a free parameter to be set in the algorithm: the number of transcripts *k *for a particular gene. In this case, this number is the internal dimension *k *of the factorization. This problem is indeed a 'dimensionality reduction problem', that is, how many dimensions explain the behavior of a set of data. In this work we have tried previous algorithms to select this number (cophenetic correlation coefficients [25,26] and SVD-based selection [27]). Cophenetic coefficients, although promising at first, did not work as expected and only gave good results when *k *is very small (one or two transcripts).

We have used a variation of the method proposed by Zhu and Ghodsi [28] (also used for NMF by Fogel *et al*. [29]). Intuitively, the idea of the algorithm is to compare how the error decreases compared with random data. If the improvement is not larger than what is obtained with random data, then there is no need to add a new transcript.

Zhu and Ghodsi [28] propose to select the dimension that maximizes a likelihood function of the singular values. The intuitive idea of their algorithm is to find the gap in the scree plot of the singular values. In this algorithm, the singular values are assumed to belong to two groups with different means and the same variance and a maximum likelihood criteria (MLC) finds the most likely partition of these groups.

In NMF there are no singular values associated with the rows and columns of matrices *W *and *H*, but the increment in the error function can be interpreted as the variation explained by the additional dimension. The input vector to the MLC is calculated as follows.

Let *Obj*_*l *_be the objective function of the factorization selecting *l *transcripts

*Obj*_*l *_= *D*_Ko_(*Y, W*_*il*_, *H*_*lk*_)

where *D*_Ko _is the Kompass divergence among *Y*, *W *and *H*.

Let $\bar{Obj_{l}}$ be the objective function of the factorization selecting *l *transcripts

$$\bar{Obj_{l}}=D_{\text{Ko}}(\hat{Y},{\hat{W}}_{il},{\hat{H}}_{lk})$$

where $\hat{Y}$ is a shuffled version of the *Y *data matrix (data for each row is randomized) and $\bar{W}$ and $\bar{H}$ are the results of the optimization.

Let *FC*_*l *_be the natural logarithm of the fold change between the objective function for *l *transcripts and the objective function for *l *- 1 transcripts:

$$FC_{l}=\log\left(\frac{Obj_{l}}{Obj_{l-1}}\right)$$

and, for convenience, it is assumed that

*Obj*_∅ _= *D*_Ko_(*Y*, *R*)

where *R *is a positive random matrix with unit variance. On the other hand

$${\bar{FC}}_{l}=\log\left(\frac{{\bar{Obj}}_{l}}{{\bar{Obj}}_{l-1}}\right)$$

We consider

$$\Delta l=FC_{l}-{\bar{FC}}_{l}$$

that is, how much the error function diminishes compared with random data. To improve the normality of the data (needed for MLC), a Box-Cox [30] transformation is performed on this vector to have standard kurtosis.

Intuitively Δ*l *will be large if the dimension is smaller than the real number of transcripts. In this case, adding a new transcript diminishes the error more than what would be expected for random data.

Finally, the MLC algorithm will find the gap in the Δ*l *that splits the dimensions in two groups: true transcripts and noise.

## Abbreviations

AS, alternative splicing; EST, expressed sequence tag; MAE, mean absolute error; MLC, maximum likelihood criteria; MM, mismatch; NMF, non-negative matrix factorization; PM, perfect match; pre-mRNA, precursor mRNA; RT-PCR, reverse transcription-polymerase chain reaction; SPACE, splicing prediction and concentration estimation; SVD, singular value decomposition.

## Authors' contributions

MAA developed the programs to perform the simulations with the synthetic dataset and the analysis of the real datasets. DG, EG and VS developed the database to retrieve the data from the Johnson experiment. PCS and APM developed the algorithm to predict the internal dimension of the factorization and performed the simulations using previous algorithms. RP and LMM provided biological insight to the project since its inception and selected the genes and their structures for the analysis. AR conceived the idea, supervised the project and developed the algorithm to predict the internal dimension of the factorization and performed the simulations using previous algorithms. All of the authors participated in the redaction, read and approved the final manuscript.

## Additional data files

The following additional data are available. Additional data file 1 contains 22 figures (S1 to S22). Figures S1-S8 show the structure of all genes and transcripts, as well as probe positions, that have been used to make the synthetic data in the simulations for the eight selected genes with SPACE algorithm. Figure S9 shows an example of the affinity, property and concentration matrices according to Wang *et al.*'s model. Figure S10 shows the results of applying SPACE algorithm to six arrays with two isoforms of CASP2 gene. Three of these simulated arrays had one concentration ratio and the other three the opposite ratio. Figures S11-S22 show the results obtained in the simulations performed for 100 random genes selected from the human genome. Additional data file 2 contains the Matlab code of the SPACE algorithms. This zipped file includes the Matlab data files (.mat) for the three genes in Johnson *et al.*'s study and the code for the algorithms to predict number of transcripts, concentrations and structure. A convenient demo script file (demojohnson.m) is included to show how to use the functions.

## Supplementary Material

Additional data file 1 Figures S1-S8 show the structure of all genes and transcripts, as well as probe positions, that have been used to make the synthetic data in the simulations for the eight selected genes with SPACE algorithm. Figure S9 shows an example of the affinity, property and concentration matrices according to Wang *et al.*'s model. Figure S10 shows the results of applying SPACE algorithm to six arrays with two isoforms of CASP2 gene (synthetic data). Three of these simulated arrays had one concentration ratio and the other three the opposite ratio. Figures S11-S22 show the results obtained in the simulations done for 100 random genes selected from the human genome (synthetic data). Click here for file

Additional data file 2 This zipped file includes the Matlab data files (.mat) for the three genes in Johnson *et al.*'s study and the code for the algorithms to predict number of transcripts, concentrations and structure. A convenient demo script file (demojohnson.m) is included to show how to use the functions. Click here for file
