# Supplementary material for: SPACE: an algorithm to predict and quantify alternatively spliced isoforms using microarrays
Source: Genome Biol. 2008 Feb 29;9(2):R46. doi: 10.1186/gb-2008-9-2-r46 (PMC2374713; doi:10.1186/gb-2008-9-2-r46)
Supplement: Additional data file 1 — Figures S1-S8 show the structure of all genes and transcripts, as well as probe positions, that have been used to make the synthetic data in the simulations for the eight selected genes with SPACE algorithm. Figure S9 shows an example of the affinity, property and concentration matrices according to Wang et al.'s model. Figure S10 shows the results of applying SPACE algorithm to six arrays with two isoforms of CASP2 gene (synthetic data). Three of these simulated arrays had one concentration ratio and the other three the opposite ratio. Figures S11-S22 show the results obtained in the simulations done for 100 random genes selected from the human genome (synthetic data). [file gb-2008-9-2-r46-S1.pdf]

Additional file 1. Gene structures used to make the synthetic data, splicing matrix model example, splicing prediction with six arrays and two opposite conditions and simulation results for 100 random genes (synthetic data)

This file contains twenty-two figures (S1 to S22). Figures S1 to S8 show the structure of all genes and transcripts, as well as probe positions, that have been used to make the synthetic data in the simulations for the 8 selected genes with SPACE algorithm. Figure S9 shows an example of the affinity, property and concentration matrices according to Wang's model. Figure S10 shows the results of applying SPACE algorithm to six arrays with two isoforms of CASP2 gene (SYNTHETIC DATA). Three of these simulated arrays had one concentration ratio and the other three the opposite ratio. Figures S11 to S22 show the results obtained in the simulations done for 100 random genes selected from the human genome (SYNTHETIC DATA).

Gene structures used to make the synthetic data

CASP2 gene

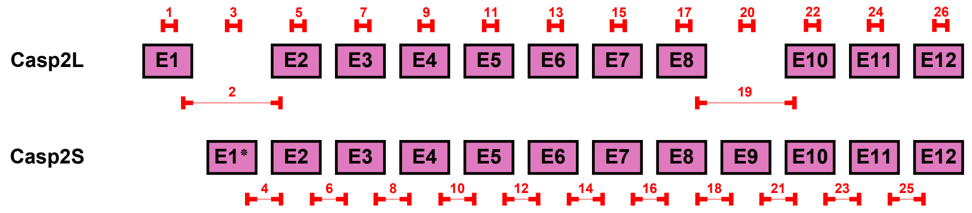

Figure S1: Structure of the different transcripts of caspase 2 (CASP2) gene and probe locations used to make the synthetic data

HNRPA2B1 gene

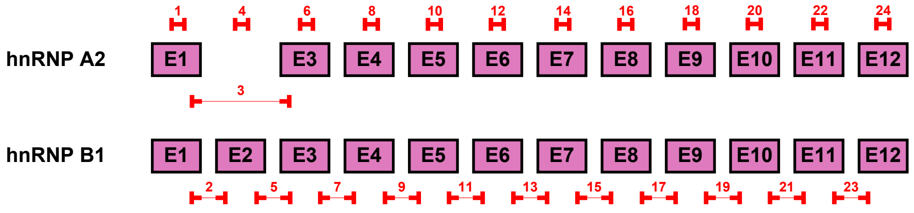

Figure S2: Structure of the different transcripts of heterogeneous nuclear ribonucleoproteins A2/B1 (HNRNP A2 / HNRNP B1) gene and probe locations used to make the synthetic data

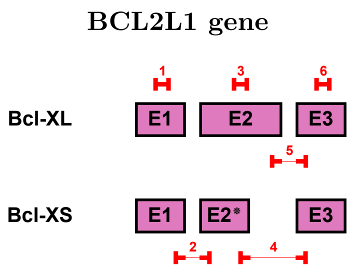

Figure S3: Structure of the different transcripts of apoptosis regulator Bcl-X (BCL-2-Like 1 protein) gene and probe locations used to make the synthetic data

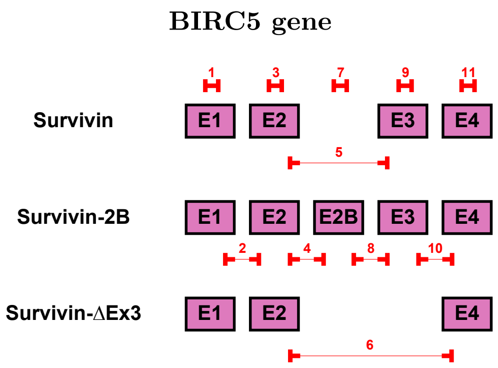

Figure S4: Structure of the different transcripts of apoptosis inhibitor survivin (BIRC5) gene and probe locations used to make the synthetic data

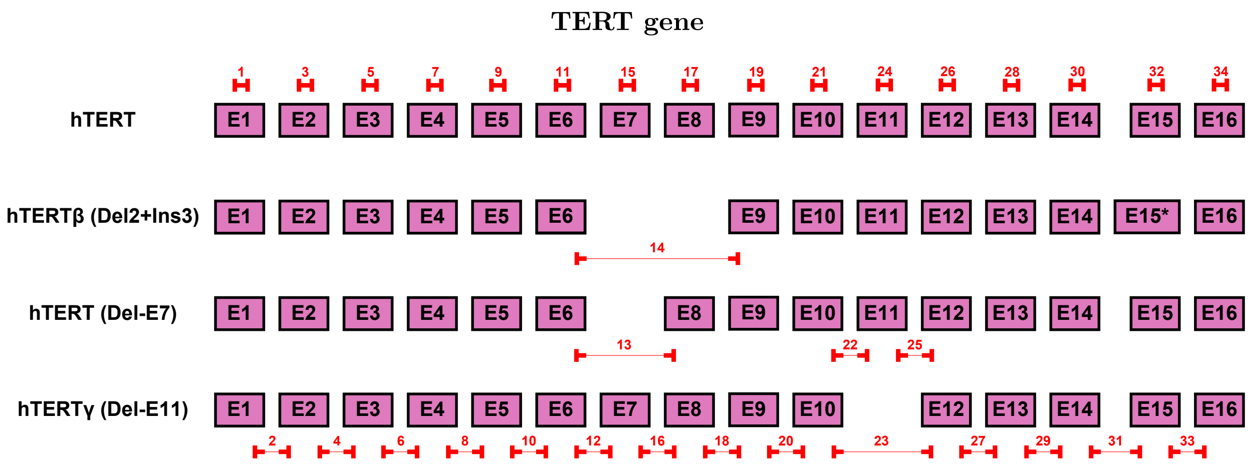

Figure S5: Structure of the different transcripts of telomerase reverse transcriptase (TERT) gene and probe locations used to make the synthetic data

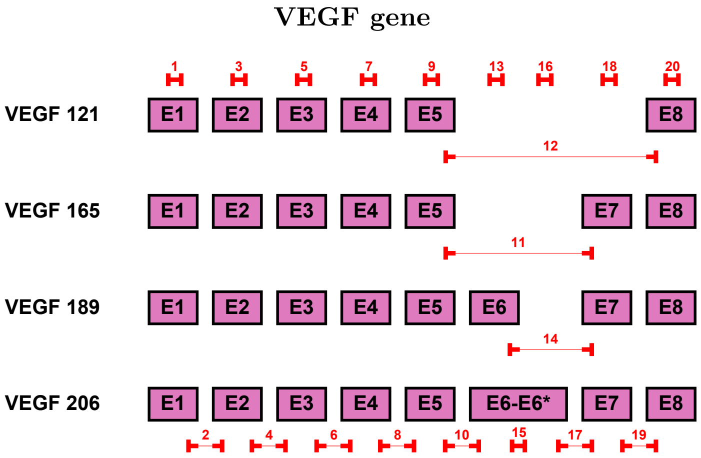

Figure S6: Structure of the different transcripts of vascular endothelial growth factor (VEGF) gene and probe locations used to make the synthetic data

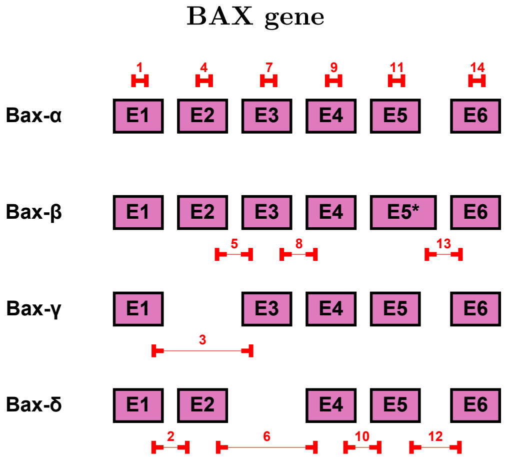

Figure S7: Structure of the different transcripts of Bcl2-associated X (BAX) protein gene and probe locations used to make the synthetic data

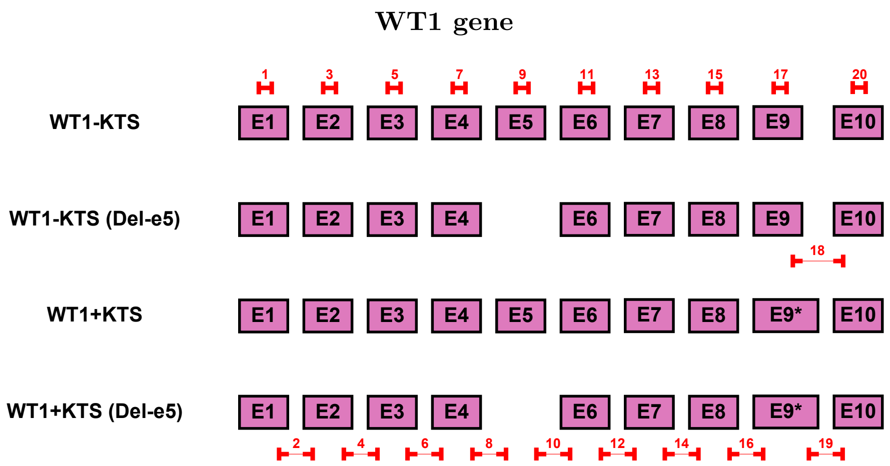

Figure S8: Structure of the different transcripts of wilms tumor 1 (WT1) gene and probe locations used to make the synthetic data

Example of building splicing matrixes in an experiment with 4 microarrays

A

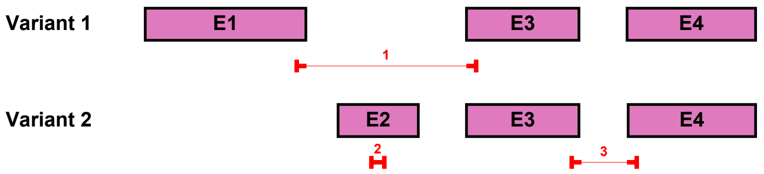

B

$$\underbrace{\begin{bmatrix} y_{11} & y_{12} & y_{13} & y_{14} \\ y_{21} & y_{22} & y_{23} & y_{24} \\ y_{31} & y_{32} & y_{33} & y_{34} \end{bmatrix}}_{\substack{\text{Probe intensities} \\ (probes \cdot arrays)}} = \underbrace{\begin{bmatrix} a_{11} & 0 & 0 \\ 0 & a_{22} & 0 \\ 0 & 0 & a_{33} \end{bmatrix}}_{\substack{\text{Probe affinities} \\ (affinities \cdot affinities)}} \underbrace{\begin{bmatrix} 1 & \alpha \\ 0 & 1 \\ 1 & 1 \end{bmatrix}}_{\substack{\text{G matrix} \\ (probes \cdot transcripts)}} \underbrace{\begin{bmatrix} t_{11} & t_{12} & t_{13} & t_{14} \\ t_{21} & t_{22} & t_{23} & t_{24} \end{bmatrix}}_{\substack{\text{Transcript concentrations} \\ (transcripts \cdot arrays)}} + \underbrace{E}_{\substack{\text{Error} \\ \text{term}}}$$

$$Y = A \;\; G \;\; T + E$$

C

$$Y \approx \begin{bmatrix} a_{11} & 0 & 0 \\ 0 & a_{22} & 0 \\ 0 & 0 & a_{33} \end{bmatrix} \begin{bmatrix} 1 & \alpha \\ 0 & 1 \\ 1 & 1 \end{bmatrix} \begin{bmatrix} t_{11} & t_{12} & t_{13} & t_{14} \\ t_{21} & t_{22} & t_{23} & t_{24} \end{bmatrix} = \underbrace{\begin{bmatrix} a_{11} & \alpha\, a_{11} \\ 0 & a_{22} \\ a_{33} & a_{33} \end{bmatrix}}_{W} \underbrace{\begin{bmatrix} t_{11} & t_{12} & t_{13} & t_{14} \\ t_{21} & t_{22} & t_{23} & t_{24} \end{bmatrix}}_{H}$$

$$Y \approx W \;\; H$$

Figure S9: Example of the splicing matrix model of a gene. (**A**) Structure of a gene with two transcripts and positions of three probes. (**B**) Mathematical model using matrixes that relates intensity of probes with structure and concentration of transcripts (proposed by Wang *et al*). Y is the matrix of microarrays measures, A is the affinity matrix, G is the property matrix which maps probes to transcripts (1 indicates perfect hybridization, 0 no hybridization and $\alpha \in [0, 1]$ indicates partial hybridization of the probe against the corresponding transcript) and T is the matrix of transcript concentrations. (**C**) If the error is low, Y matrix can be approximated by the product of two non-negative matrices W and H. The maximum value of each row of the W matrix is the affinity of the corresponding probe.

Splicing prediction with six arrays and two opposite conditions

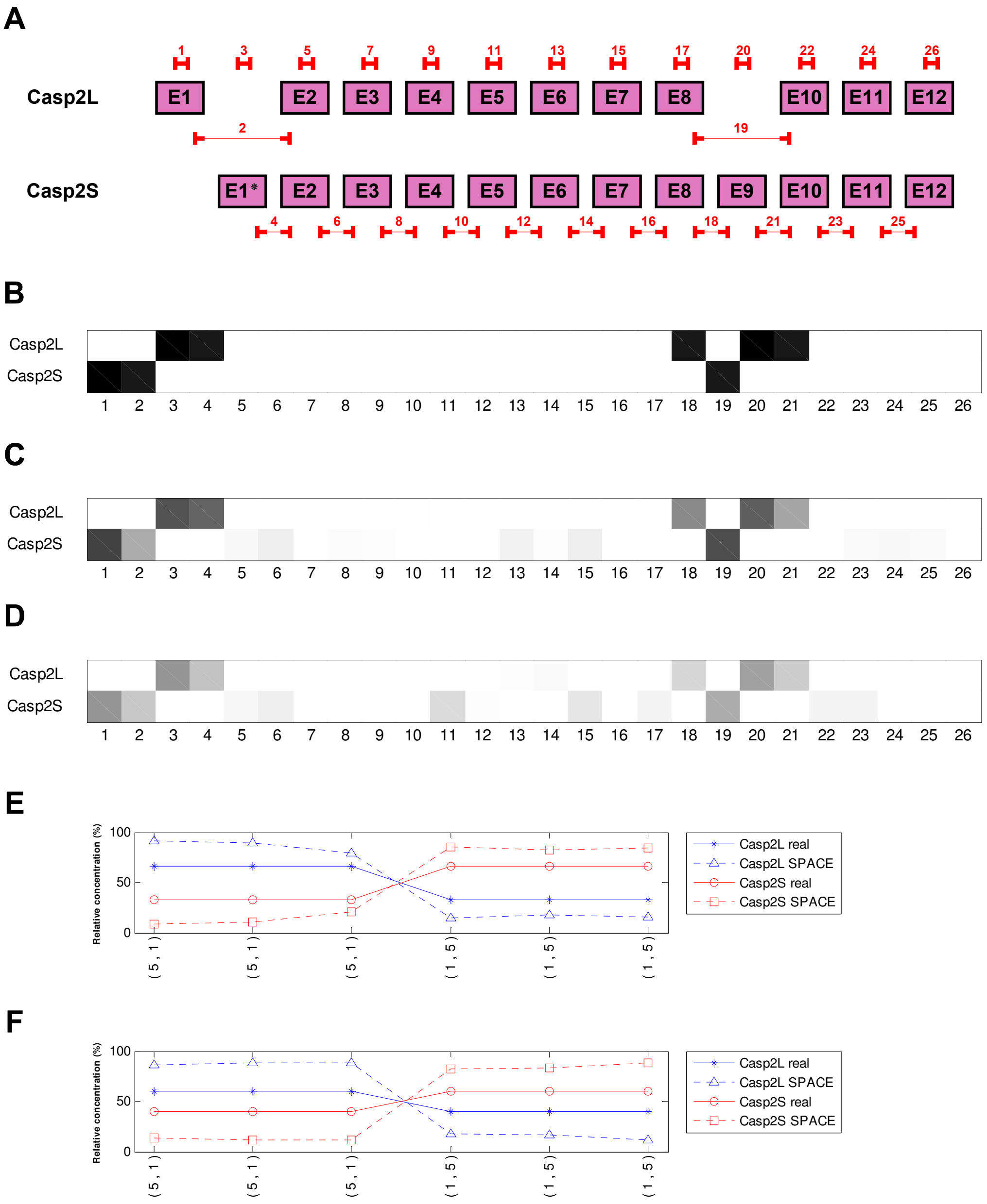

Figure S10: Experiment done with CASP2 gene (transcripts Casp2L and Casp2S). Three arrays were performed with a particular concentration ratio between its two isoforms and another three with the opposite ratio. The overall concentration of the gene were kept constant (SYNTHETIC DATA). (**A**) Structure of the two transcripts of CASP2 gene and location of probes in the microarray. (**B**) Real structure of CASP2 gene indicated by probes. Probes that match perfectly are represented by white color (100%), no hybridization by black color (0%) while partial hybridization by different shades of gray. (**C**) Predicted splicing structure with an alternating concentration ratio equal to 2:1. Compared with the real structure of transcripts (panel B), the predicted one still keeps strong similarity. (**D**) Predicted splicing structure with an alternating concentration ratio equal to 1.5:1. Comparing with the real structure (panel B) and former prediction (panel C), it can be observed that the accuracy of splicing structure prediction decreases as concentration ratio approaches to 1:1. (**E**) Real and estimated relative concentrations with an alternating concentration ratio of 2:1. (**F**) Real and estimated relative concentrations with an alternating concentration ratio of 1.5:1.

Simulation results for 100 random genes (synthetic data)

Table 1: Simulation done for 100 random genes selected from the human genome with 2 to 5 transcripts. Genes with same number of different transcripts and similar number of exons have been grouped together. In the table below, the number of genes inside each respective group is shown as well as a reference to the corresponding figure.

	**Number of Exons**			
**Number of Transcripts**	**2 to 5**	**6 to 10**	**11 to 20**	**more than 20**
**2**	15<br>Figure:S11	23<br>Figure:S12	15<br>Figure:S13	5<br>Figure:S14
**3**	0	7<br>Figure:S15	5<br>Figure:S16	10<br>Figure:S17
**4**	0	3<br>Figure:S18	2<br>Figure:S19	0
**5**	0	3<br>Figure:S20	8<br>Figure:S21	4<br>Figure:S22

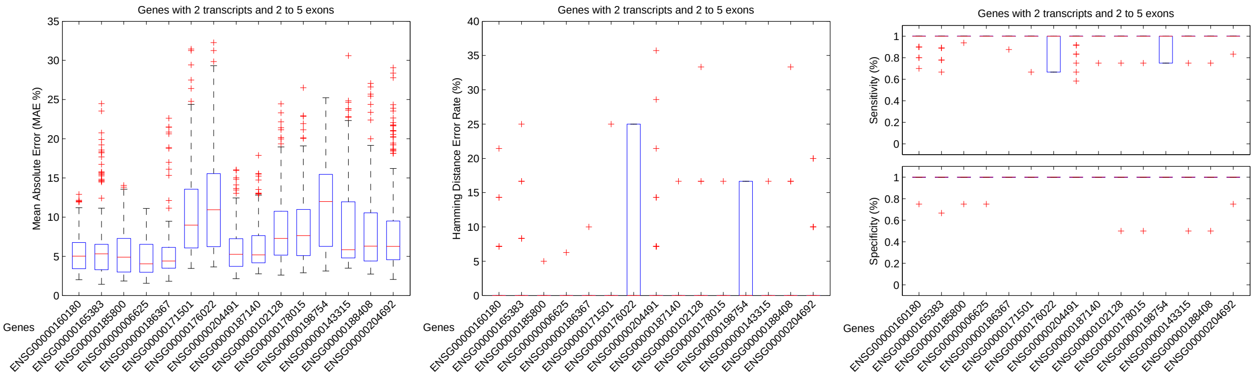

(a) MAE (b) Hamming Distance (c) Sensibility and specificity

Figure S11: Simulation results of genes with 2 transcripts and 2 to 5 exons. In this and in the following figures, genes are sorted by decreasing number of probes with different hybridization patterns and decreasing number of exons. The hybridization pattern is defined as the binding capability of a probe with each of the transcripts of a gene, i.e., a logical vector that shows whether the probe belongs to each transcript or not. It is expected that structure predictive accuracy increases by increasing the number of probes with different hybridization patterns. In the figures above, ENSG00000171501, ENSG00000176022 and ENSG00000198754 genes present a higher error and seem not to follow the former rule. These genes have two transcripts of only one exon and they share most of their sequence, this feature makes it difficult to distinguish between them. In addition, the number of probes of these genes is very small (2 or 3).

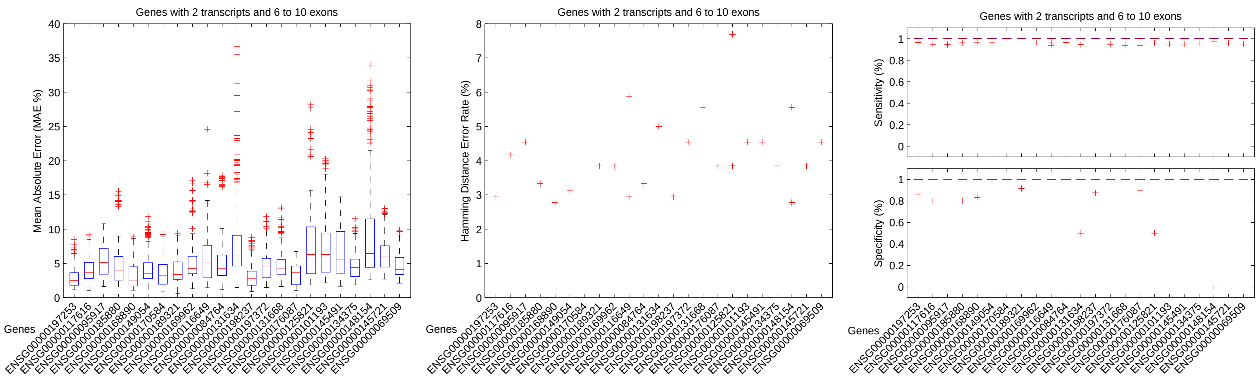

(a) MAE (b) Hamming Distance (c) Sensibility and specificity

Figure S12: Simulation results of genes with 2 transcripts and 6 to 10 exons. ENSG00000131634 and ENSG00000148154 genes seem to give more outlier results in the concentration estimation than the other genes. These genes have two very similar transcripts which share most of their sequence. In the simulations done, only one probe is able to discern between them. If this probe is of bad quality (low affinity) in the corresponding simulated test, accuracy decreases.

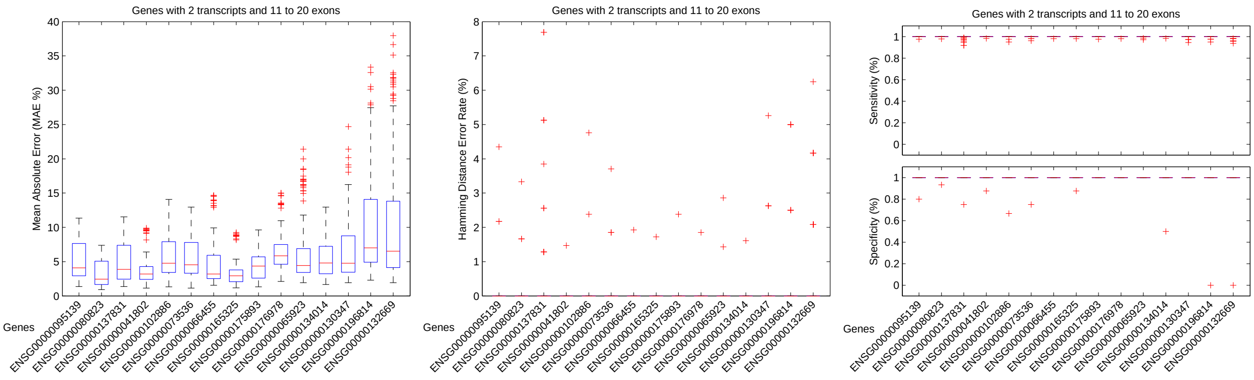

(a) MAE (b) Hamming Distance (c) Sensibility and specificity

Figure S13: Simulation results of genes with 2 transcripts and 11 to 20 exons. As in figure S12, ENSG00000131634 and ENSG00000148154 genes seem to give more outlier results in the concentration estimation than the other genes. These genes have two transcripts that only differ in the length of the last exon with only one different probe that discerns between them.

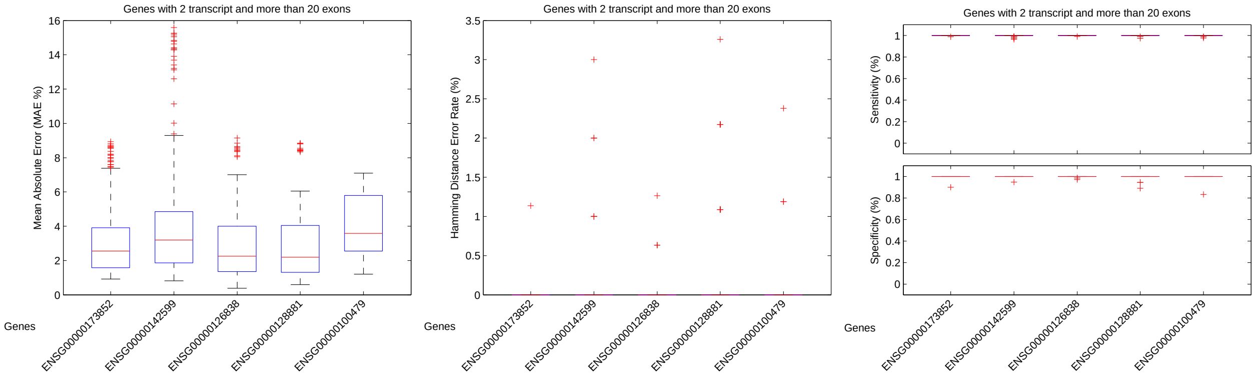

(a) MAE (b) Hamming Distance (c) Sensibility and specificity

Figure S14: Simulation results of genes with 2 transcripts and more than 20 exons. Errors are low in this case since the number of probes per gene is large.

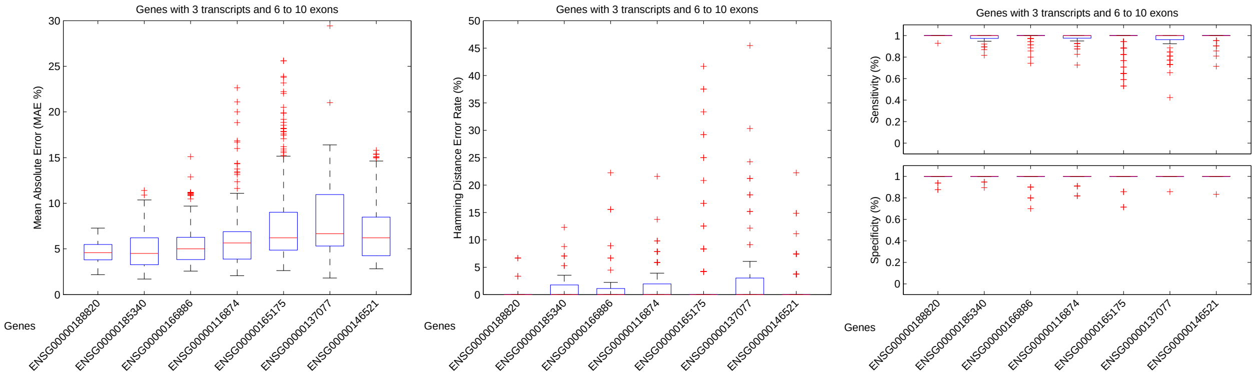

(a) MAE (b) Hamming Distance (c) Sensibility and specificity

Figure S15: Simulation results of genes with 3 transcripts and 6 to 10 exons. ENSG00000165175 gene has worse accuracy both in concentration estimation an in structure prediction than the other genes. For this gene, only one probe can discriminate between two of its three transcripts making the quality of that probe critical in order to obtain good results.

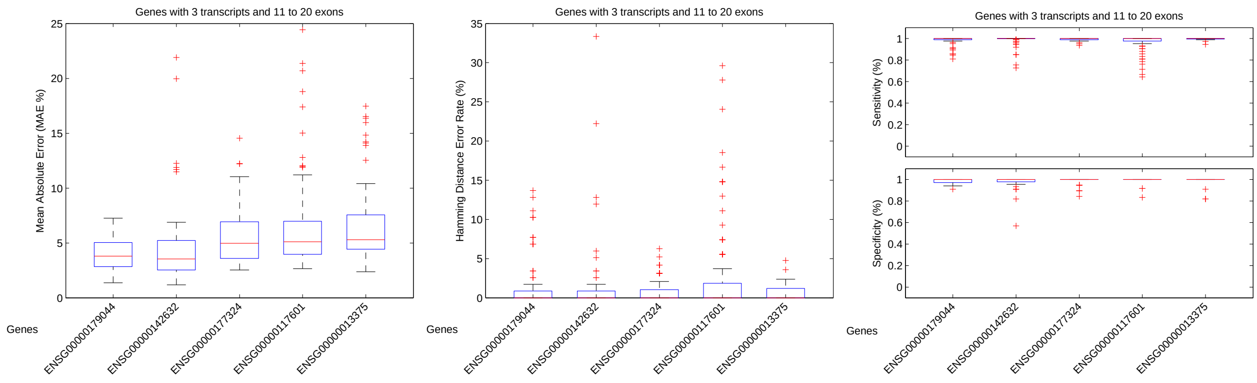

(a) MAE (b) Hamming Distance (c) Sensibility and specificity

Figure S16: Simulation results of genes with 3 transcripts and 11 to 20 exons. ENSG00000117601 gene does not differ very much from the other genes but it has slightly more outliers. This can be the effect of having two of its three transcripts with almost the same sequence.

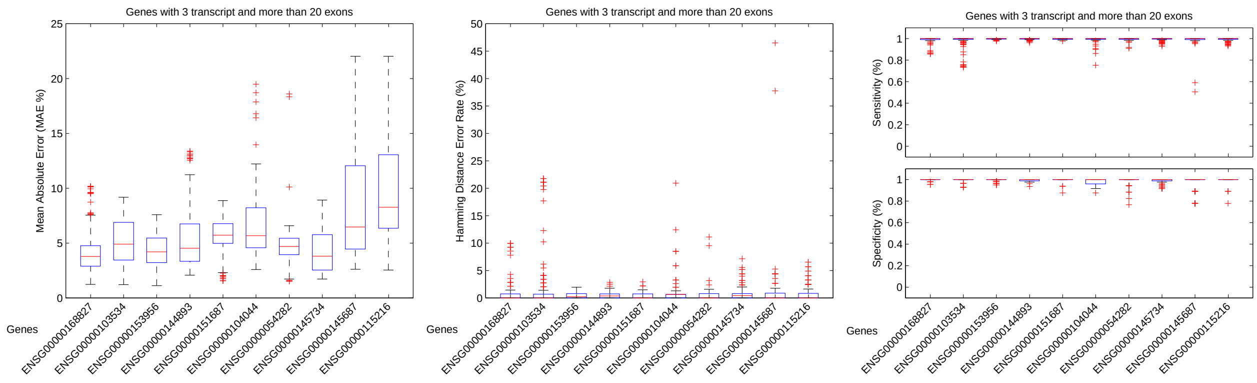

(a) MAE (b) Hamming Distance (c) Sensibility and specificity

Figure S17: Simulation results of genes with 3 transcripts and more than 20 exons. Results are similar to that obtained in the previous figures. Performance decreases as the number of discriminative probes decreases.

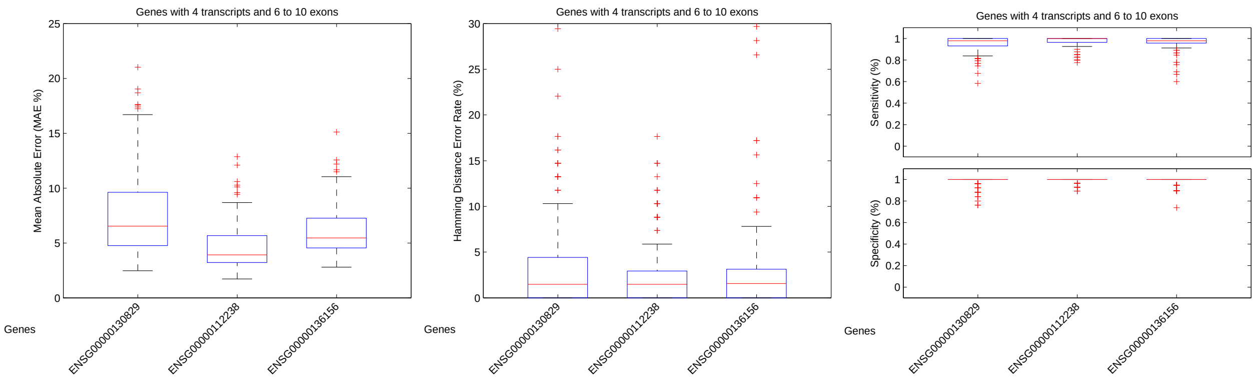

(a) MAE (b) Hamming Distance (c) Sensibility and specificity

Figure S18: Simulation results of genes with 4 transcripts and 6 to 10 exons. ENSG00000130829 has more error than the other genes due to having two of its transcripts almost identical (differ only in seven nucleotides).

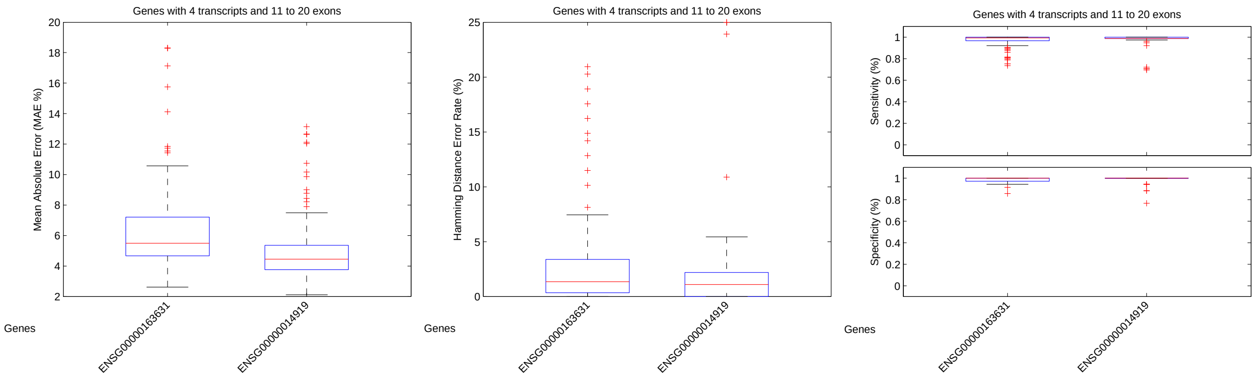

(a) MAE (b) Hamming Distance (c) Sensibility and specificity

Figure S19: Simulation results of genes with 4 transcripts and 11 to 20 exons. In this simulation in spite of the fact that ENSG0000163631 gene has more probes with different hybridization pattern than ENSG00000014919 gene the former has more error probably due to the fact that two of its transcripts share almost all sequence an differ only in one probe.

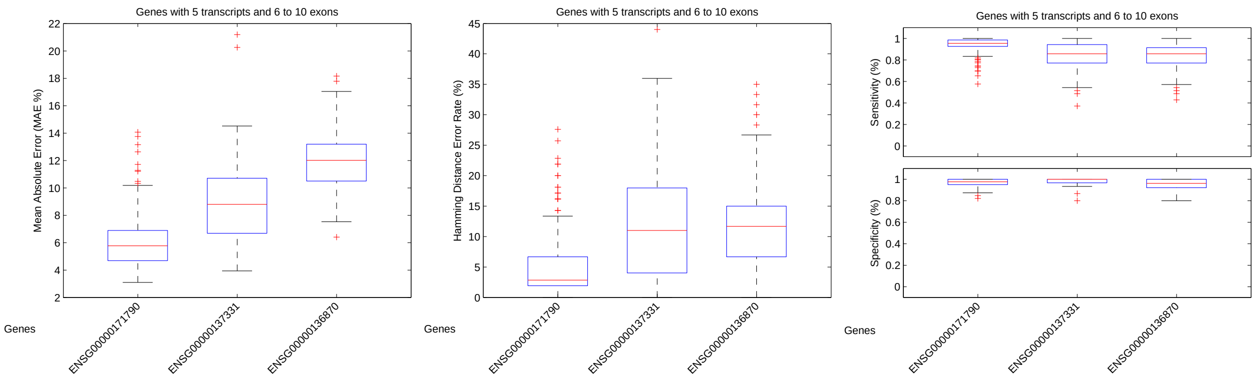

(a) MAE (b) Hamming Distance (c) Sensibility and specificity

Figure S20: Simulation results of genes with 5 transcripts and 6 to 10 exons

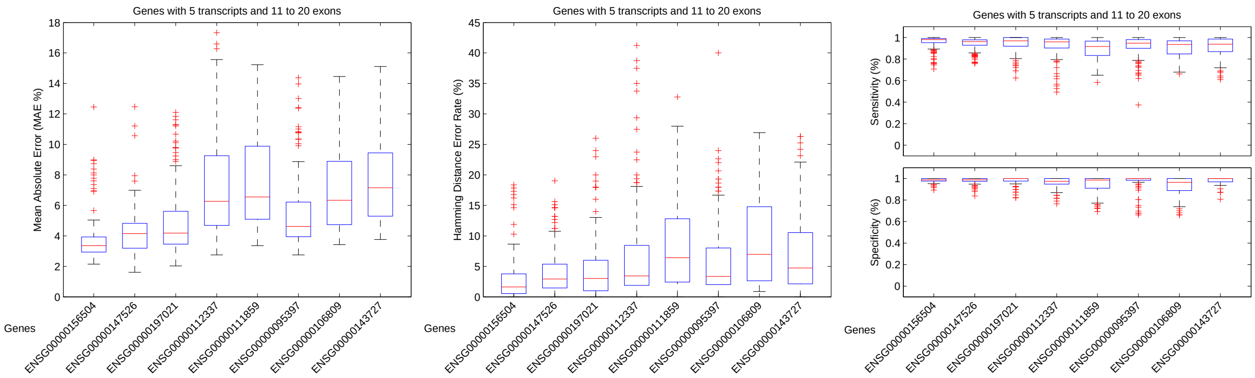

(a) MAE (b) Hamming Distance (c) Sensibility and specificity

Figure S21: Simulation results of genes with 5 transcripts and 11 to 20 exons. ENSG00000112337 and ENSG00000111859 present a high error because of having very similar transcripts. In the simulations, error increases in long genes with transcripts that differ by only one or two exons (cassette or exon-intron retention). This is due to the very low proportion of probes able to discern one transcript from another.

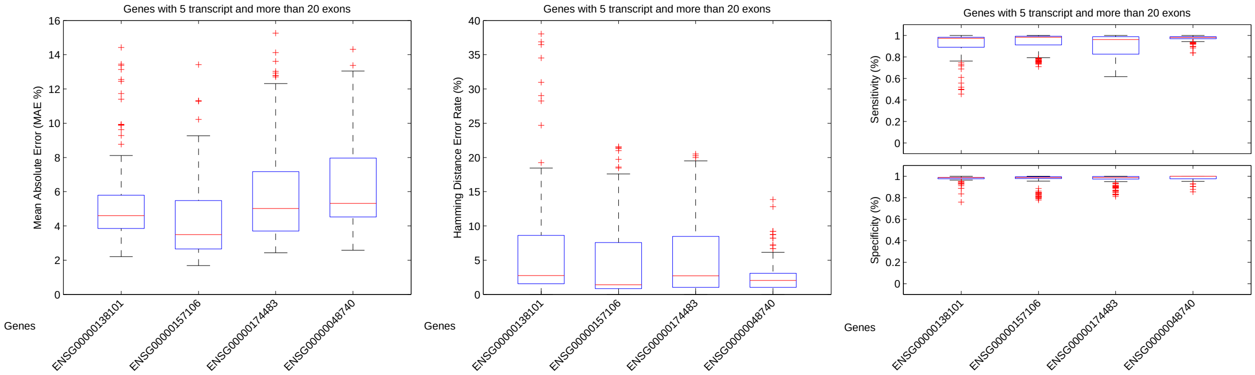

(a) MAE (b) Hamming Distance (c) Sensibility and specificity

Figure S22: Simulation results of genes with 5 transcripts and more than 20 exons
